# Supplementary material for: Carnosinase-1 Knock-Out Reduces Kidney Fibrosis in Type-1 Diabetic Mice on High Fat Diet
Source: Antioxidants (Basel). 2023 Jun 14;12(6):1270. doi: 10.3390/antiox12061270 (PMC10295340; doi:10.3390/antiox12061270)
Supplement: Supplementary file 1 [file antioxidants-12-01270-s001.zip › Suppl. figures 18-4-23.pptx]

## Slide 1
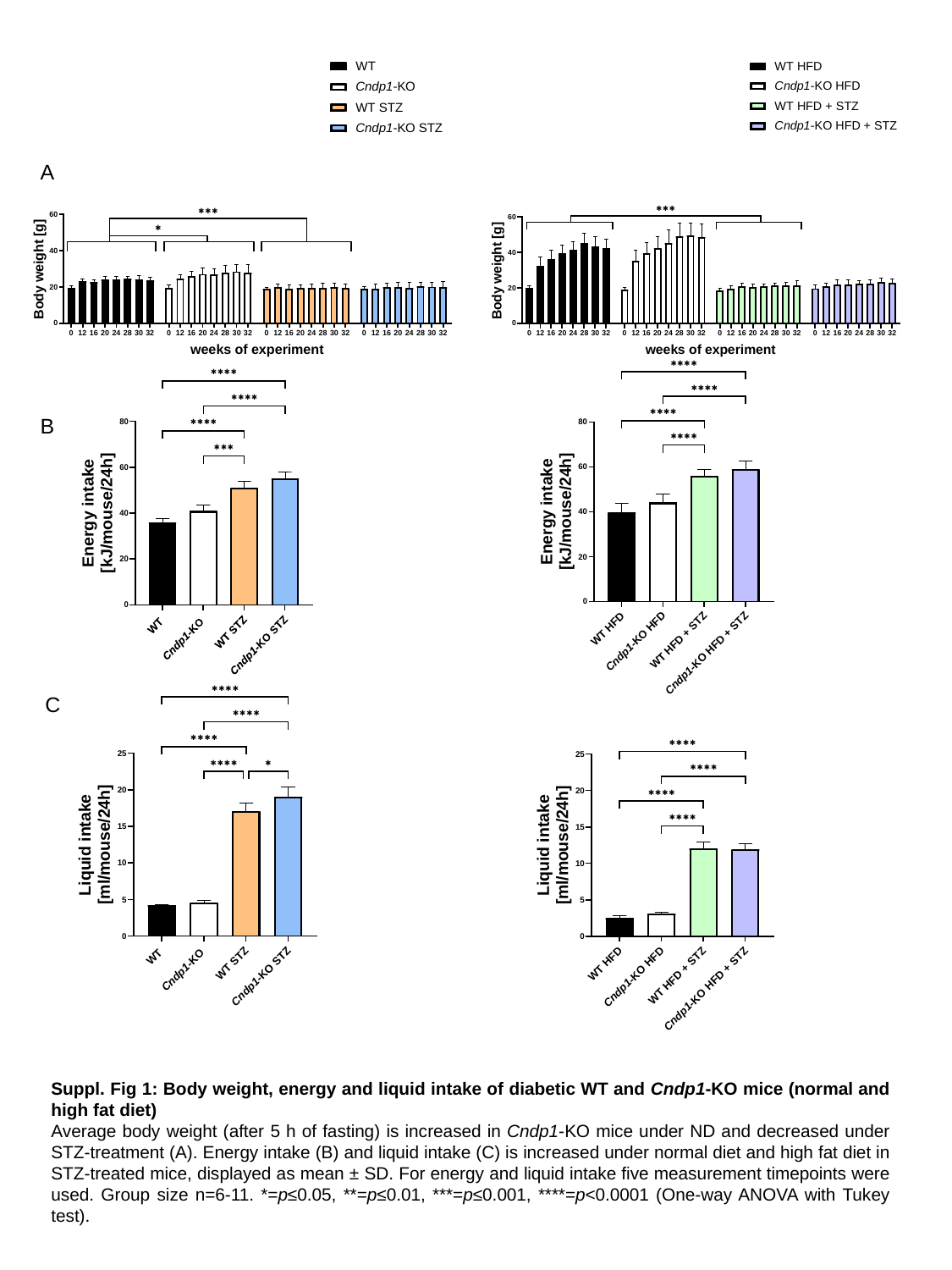

A
B
C
Suppl. Fig 1: Body weight, energy and liquid intake of diabetic WT and Cndp1-KO mice (normal and high fat diet)
Average body weight (after 5 h of fasting) is increased in Cndp1-KO mice under ND and decreased under STZ-treatment (A). Energy intake (B) and liquid intake (C) is increased under normal diet and high fat diet in STZ-treated mice, displayed as mean ± SD. For energy and liquid intake five measurement timepoints were used. Group size n=6-11. *=p≤0.05, **=p≤0.01, ***=p≤0.001, ****=p<0.0001 (One-way ANOVA with Tukey test).

## Slide 2
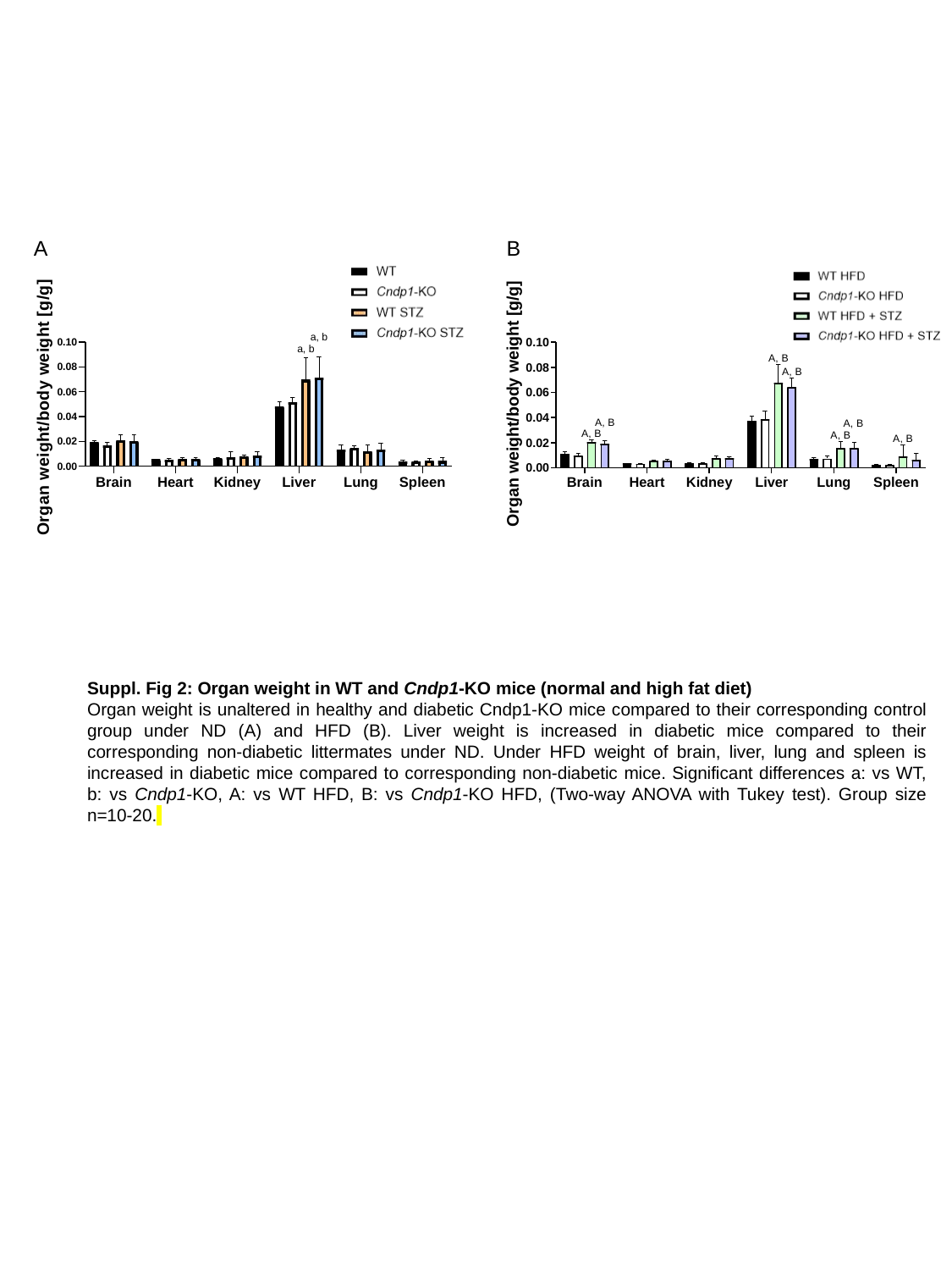

A
B
Suppl. Fig 2: Organ weight in WT and Cndp1-KO mice (normal and high fat diet)
Organ weight is unaltered in healthy and diabetic Cndp1-KO mice compared to their corresponding control group under ND (A) and HFD (B). Liver weight is increased in diabetic mice compared to their corresponding non-diabetic littermates under ND. Under HFD weight of brain, liver, lung and spleen is increased in diabetic mice compared to corresponding non-diabetic mice. Significant differences a: vs WT, b: vs Cndp1-KO, A: vs WT HFD, B: vs Cndp1-KO HFD, (Two-way ANOVA with Tukey test). Group size n=10-20.

## Slide 3
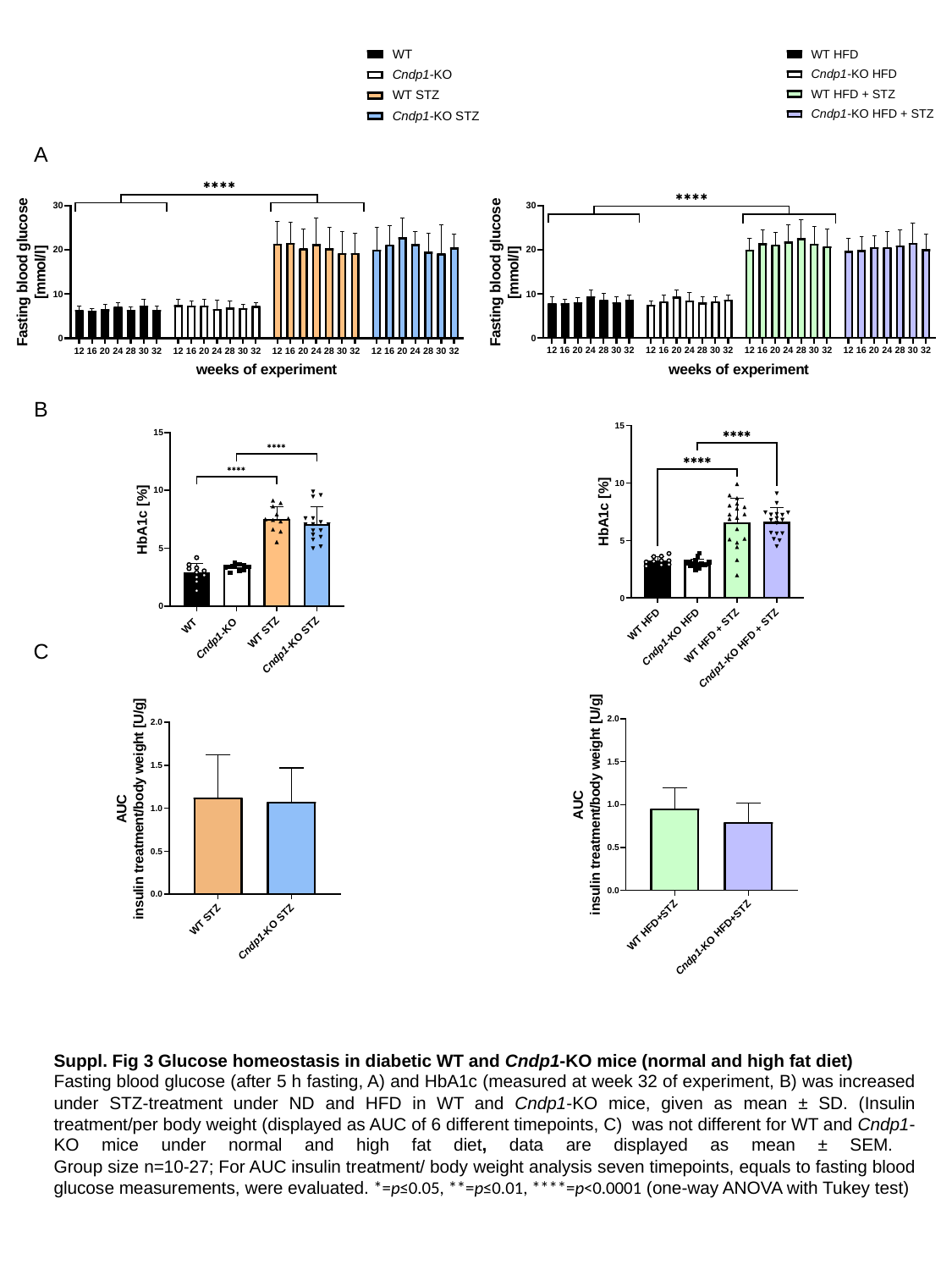

A
B
C
Suppl. Fig 3 Glucose homeostasis in diabetic WT and Cndp1-KO mice (normal and high fat diet)
Fasting blood glucose (after 5 h fasting, A) and HbA1c (measured at week 32 of experiment, B) was increased under STZ-treatment under ND and HFD in WT and Cndp1-KO mice, given as mean ± SD. (Insulin treatment/per body weight (displayed as AUC of 6 different timepoints, C) was not different for WT and Cndp1-KO mice under normal and high fat diet, data are displayed as mean ± SEM.
Group size n=10-27; For AUC insulin treatment/ body weight analysis seven timepoints, equals to fasting blood glucose measurements, were evaluated. *=p≤0.05, **=p≤0.01, ****=p<0.0001 (one-way ANOVA with Tukey test)

## Slide 4
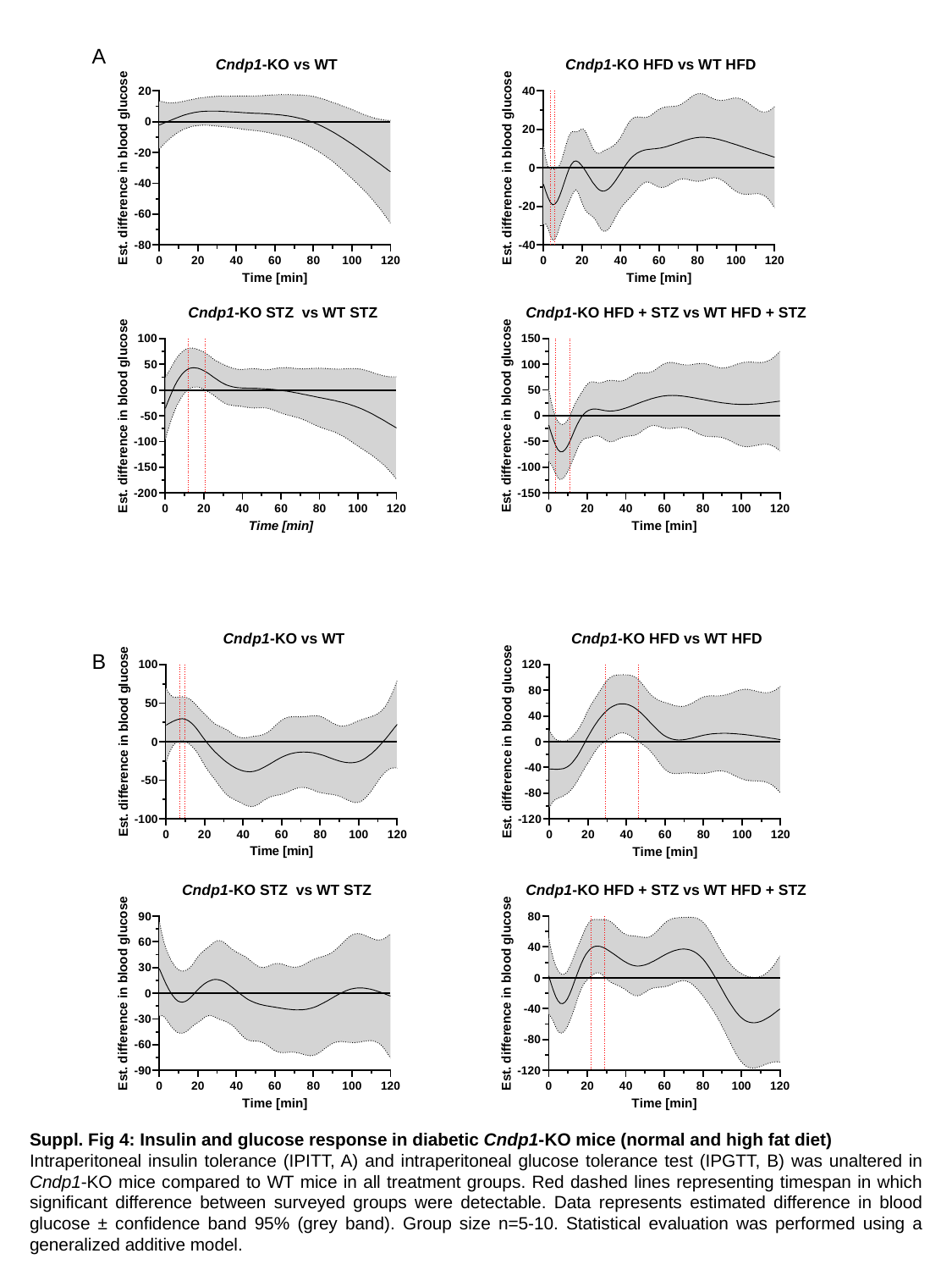

A
B
Suppl. Fig 4: Insulin and glucose response in diabetic Cndp1-KO mice (normal and high fat diet)
Intraperitoneal insulin tolerance (IPITT, A) and intraperitoneal glucose tolerance test (IPGTT, B) was unaltered in Cndp1-KO mice compared to WT mice in all treatment groups. Red dashed lines representing timespan in which significant difference between surveyed groups were detectable. Data represents estimated difference in blood glucose ± confidence band 95% (grey band). Group size n=5-10. Statistical evaluation was performed using a generalized additive model.

## Slide 5
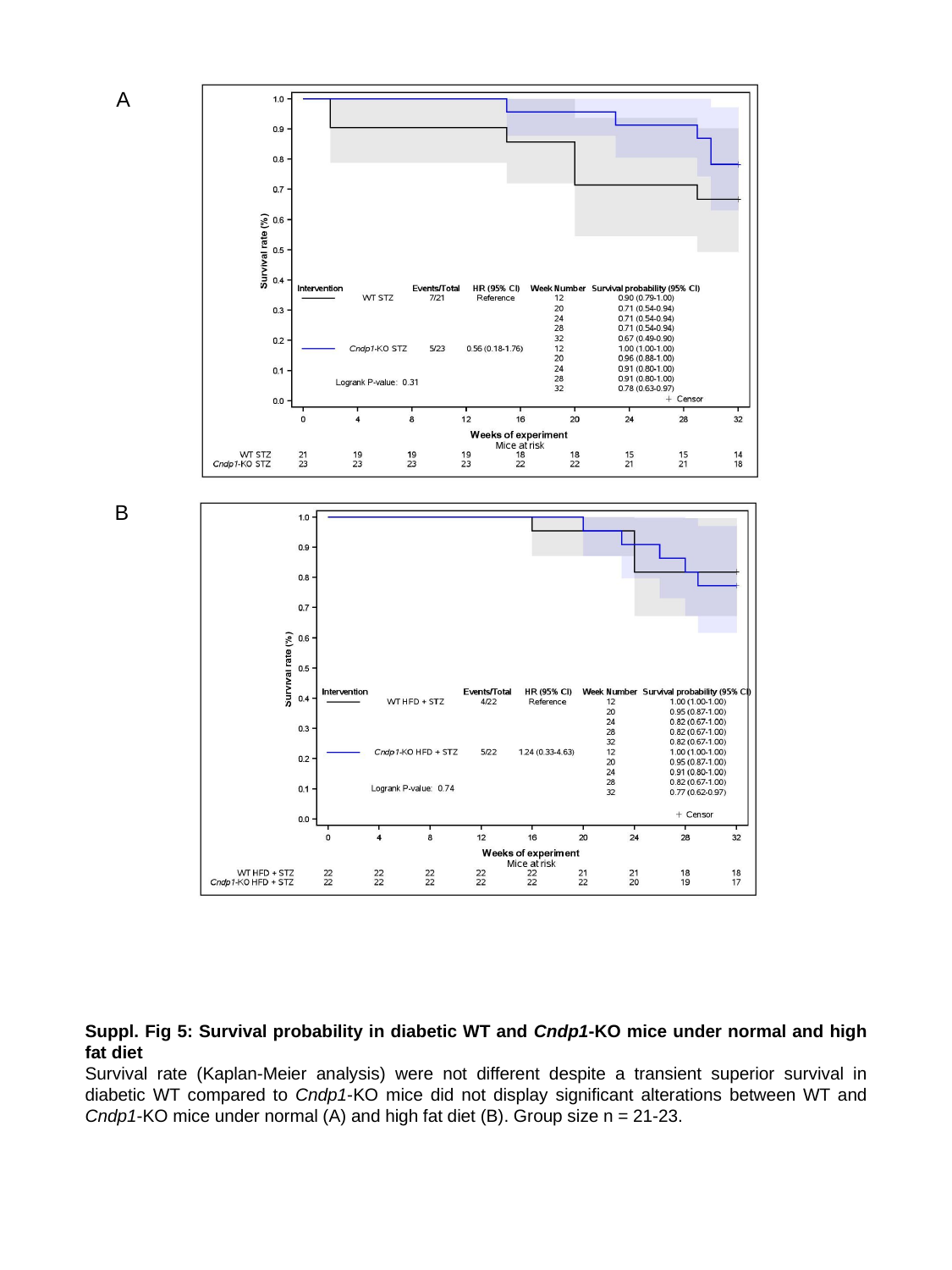

A
B
Suppl. Fig 5: Survival probability in diabetic WT and Cndp1-KO mice under normal and high fat diet
Survival rate (Kaplan-Meier analysis) were not different despite a transient superior survival in diabetic WT compared to Cndp1-KO mice did not display significant alterations between WT and Cndp1-KO mice under normal (A) and high fat diet (B). Group size n = 21-23.
