# Supplementary material for: Carnosinase-1 Knock-Out Reduces Kidney Fibrosis in Type-1 Diabetic Mice on High Fat Diet
Source: Antioxidants (Basel). 2023 Jun 14;12(6):1270. doi: 10.3390/antiox12061270 (PMC10295340; doi:10.3390/antiox12061270)
Supplement: Supplementary file 1 [file antioxidants-12-01270-s001.zip › Suppl. Table 9.pdf]

Suppl. Table 9: Metabolomic profile in kidney

9.1 Normal diet

class	Metabolite	WT	Cndp1-KO	*p* (vs WT)	WT STZ	Cndp1-KO STZ	*p* (vs WT STZ)
**Amino acid**	Alanine	476±34	519.8±44.5	0.116	469±76.7	573±193.4	0.29
**Amino acid**	Arginine	76.1±5.4	75.4±17.5	0.94	117.7±38.7	122.8±21.2	0.805
**Amino acid**	Asparagine	57.2±8.0	519.8±44.5	0.78	60.2±13.8	73.0±16.1	0.206
**Amino acid**	Aspartic acid	592.6±71.9	75.4±17.5	0.04*	417.9±139.2	458.5±227.7	0.741
**Amino acid**	Glutamine	340.7±74.6	58.9±10.3	0.678	405.7±105.5	406.1±108.5	0.996
**Amino acid**	Glutamate	2243.7±309.7	474.3±85.3	0.781	1818.2±89.9	1931.1±316.8	0.462
**Amino acid**	Glycine	1993.8±323.1	360.2±70.0	0.909	1291.3±109.5	1578.3±278.2	0.058
**Amino acid**	Histidine	148.9±43.8	2186.9±317.2	0.086	171.4±53.1	167.9±44.8	0.914
**Amino acid**	Isoleucine	63.7±16.2	1967.3±383.2	0.532	85.2±19.2	93.6±16.5	0.473
**Amino acid**	Leucine	131.2±19.1	108.3±18.5	0.332	191.4±49.9	193.3±36.0	0.949
**Amino acid**	Lysine	110.4±16.2	69.4±11.4	0.549	82.6±12.1	108.9±26.2	0.069
**Amino acid**	Methionine	65.0±2.6	144.3±21.4	0.013*	49.9±12.9	63.0±16.8	0.199
**Amino acid**	Phenylalanine	58.2±5.3	118.3±23.2	0.299	68.9±11.0	75.7±13.5	0.403
**Amino acid**	Proline	69.4±13.1	57.8±4.6	0.107	74.0±21.3	77.0±17.8	0.815
**Amino acid**	Serine	633.7±79.3	63.7±9.7	0.392	466.3±59.4	537.0±106.1	0.224
**Amino acid**	Tyrosine	63.1±10.2	56.3±9.9	0.548	54.5±12.7	74.2±21.1	0.105
**Amino acid**	Valine	119.1±24.3	589.1±78.0	0.811	162.1±35.6	166.7±25.1	0.817
**Acylcarnitines**	L-Carnitine	452±136.8	322.3±97.3	0.229	323.8±51.4	312.5±44.9	0.785
**Acylcarnitines**	L-Acetylcarnitine	90±17	59.2±16.9	0.067	64.2±16.7	67.5±7.9	0.772
**Acylcarnitines**	Propionylcarnitine	3.5±0.7	2.9±0.9	0.413	5.6±1.7	4±1	0.189
**Acylcarnitines**	Malonylcarnitine	0.6±0.2	0.6±0.2	0.925	1.1±0.4	0.9±0.4	0.519
**Acylcarnitines**	Butyrylcarnitine	4.6±1.3	3.7±1.6	0.465	6.3±2.6	6.5±0.8	0.889
**Acylcarnitines**	Butenylcarnitine	0.1±0.1	0.1±0.1	0.109	0.2±0.1	0.2±0.1	0.6
**Acylcarnitines**	2-Methylbutyroylcarnitine	0.6±0.2	0.5±0.3	0.768	1.2±0.7	1.1±0.4	0.889
**Acylcarnitines**	Glutarylcarnitine	0.7±0.1	0.6±0.2	0.506	0.8±0.1	0.7±0.1	0.519
**Acylcarnitines**	3-Methylglutarylcarnitine	0.2±0.1	0.2±0.1	0.771	0.2±0.1	0.2±0.1	0.909
**Acylcarnitines**	Methylmalonylcarnitine	2.1±0.3	2.1±0.7	0.972	2.5±0.8	2.3±0.7	0.841
**Acylcarnitines**	Glutaconylcarnitine	0.1±0.1	0.1±0.1	0.484	0.1±0.1	0.1±0.1	0.856
**Acylcarnitines**	Hexanoylcarnitine	1±0.2	0.8±0.2	0.309	1.1±0.3	1.2±0.3	0.76
**Acylcarnitines**	L-Octanoylcarnitine	0.2±0.2	0.2±0.2	0.936	0.2±0.2	0.3±0.2	0.491
**Acylcarnitines**	Nonanoylcarnitine	0.1±0.1	0.1±0.1	0.838	0.2±0.1	0.2±0.1	0.876
**Acylcarnitines**	Dodecanoylcarnitine	0.6±0.2	0.6±0.2	0.999	0.6±0.1	0.6±0.2	0.766
**Acylcarnitines**	Tetradecanoylcarnitine	2±0.7	1.7±0.5	0.581	1.8±0.4	2±0.4	0.4
**Acylcarnitines**	cis-5-Tetradecenoylcarnitine	0.4±0.1	0.3±0.2	0.809	0.4±0.1	0.4±0.1	0.969
**Acylcarnitines**	3-Hydroxy-cis-5-tetradecenoylcarnitine	0.2±0.1	0.1±0.1	0.355	0.2±0.1	0.2±0.1	0.67
**Acylcarnitines**	O-(hydroxytetradecadienoyl)-L-carnitine	0.1±0.1	0.1±0.1	0.414	0.1±0.1	0.1±0.1	0.905

**Acylcarnitines**	L-Palmitoylcarnitine	9.9±1.9	8.6±2.3	0.459	8.6±1.5	10.8±1.4	0.106
**Acylcarnitines**	3-Hydroxyhexadecanoylcarnitine	0.3±0.1	0.3±0.1	0.306	0.3±0.1	0.3±0.1	0.912
**Acylcarnitines**	9-Hexadecenoylcarnitine	0.8±0.2	0.8±0.3	0.804	0.9±0.3	1.1±0.3	0.335
**Acylcarnitines**	3-Hydroxy-9-hexadecenoylcarnitine	0.2±0.1	0.1±0.1	0.054	0.2±0.1	0.2±0.1	0.926
**Acylcarnitines**	9.12-Hexadecadienoylcarnitine	0.3±0.1	0.3±0.1	0.294	0.4±0.1	0.4±0.1	0.732
**Acylcarnitines**	Stearoylcarnitine	4.5±0.7	4±0.9	0.488	4.2±0.4	5.4±0.9	0.082
**Acylcarnitines**	Oleoylcarnitine	3.9±0.7	3.6±1.1	0.724	4.4±0.6	5.4±1.4	0.278
**Acylcarnitines**	3-Hydroxy-11Z-octadecenoylcarnitine	0.3±0.1	0.3±0.2	0.659	0.4±0.1	0.4±0.1	0.838
**Acylcarnitines**	Linoleyl carnitine	1.7±0.4	1.3±0.5	0.259	2.2±0.6	2.4±0.7	0.697
**Amino acid related**	1-Methylhistidine	27.1±3.6	22.9±5.3	0.303	39.3±5.5	38.5±11.1	0.92
**Amino acid related**	3-Methylhistidine	5.3±0.5	1.6±0.4	0.001*	3.9±1	2.1±0.6	0.03*
**Amino acid related**	5-Aminopentanoic acid	2.5±0.8	1.6±0.3	0.105	3.3±2.3	4.4±1.6	0.546
**Amino acid related**	L-alpha-Aminobutyric acid	24.2±6.2	40.1±8.2	0.036*§	33.3±20.7	34±11.2	0.96
**Amino acid related**	Asymmetric dimethylarginine	5±1	5.1±0.8	0.835	5±0.9	5.2±2.2	0.922
**Amino acid related**	Aminoadipic acid	38±14	23.7±7.2	0.165	24.4±10.4	55.1±41.6	0.261
**Amino acid related**	3-Aminobutanoic acid	2.4±0.9	2.9±1.1	0.462	3.2±1.1	4.1±1.9	0.48
**Amino acid related**	Betaine	1618.3±423.5	1512.5±771.1	0.842	1688.8±638.2	2894±965	0.122
**Amino acid related**	Citrulline	16.7±4.3	20.4±4.1	0.311	24.9±7.9	35±10.5	0.231
**Amino acid related**	Creatinine	25.4±4.7	33.8±14.3	0.37	24.6±5.8	37.8±10.7	0.109
**Amino acid related**	Cystine	708±519.8	553±152.3	0.638	812.8±408	1185.8±420.3	0.313
**Amino acid related**	Homo-L-arginine	0.7±0.8	0.4±0.2	0.593	0.4±0.3	0.4±0.2	0.863
**Amino acid related**	Homocysteine	52.5±18.5	62.1±11.4	0.474	46.8±11	41.4±21.7	0.718
**Amino acid related**	Ornithine	12.5±3.4	20.7±9	0.186	21.6±3.8	27.5±3.4	0.091
**Amino acid related**	Phenylacetylglycine	10.6±1.7	29.6±15.5	0.079	41.4±23.9	92.5±58.8	0.212
**Amino acid related**	L-phenylalanine betaine	0.1±0.1	0.1±0.1	0.84	0.1±0.1	0.2±0.1	0.369
**Amino acid related**	Proline betaine	16.3±2.5	11.4±3.3	0.08	6.7±3.1	18.1±4.4	0.011*§
**Amino acid related**	Sarcosine	3.8±1.2	2.1±0.7	0.075	1.8±0.2	2.9±1.1	0.106
**Amino acid related**	Symmetric dimethylarginine	0.8±0.2	0.9±0.3	0.748	0.8±0.2	1.1±0.5	0.292
**Amino acid related**	4-Hydroxyproline	26.1±4	23.5±2.5	0.365	18.4±3	20.4±4.1	0.523
**Amino acid related**	Taurine	3207.8±321.8	3122.5±400.4	0.784	3382.8±254	3472.5±257.6	0.683
**Bile acids**	Cholic acid	2.5±1.1	2.1±1.6	0.683	6.7±4.4	3.6±2	0.297
**Bile acids**	Deoxycholic acid	0.6±0.3	0.3±0.2	0.144	0.9±0.4	1±0.4	0.819
**Bile acids**	Taurocholic acid	4.8±1.9	29.2±32.9	0.246	11.1±8.3	10.5±10.8	0.942
**Bile acids**	Taurochenodesoxycholic acid	0.2±0.1	0.7±0.6	0.166	0.9±0.9	0.3±0.3	0.298
**Bile acids**	Taurodeoxycholic acid	0.3±0.1	2.2±2.4	0.22	0.6±0.6	0.5±0.4	0.836
**Bile acids**	Tauro-b-muricholic acid	1.7±0.7	7±6.3	0.192	5.5±4.6	4.2±5.9	0.777
**biogenic amines**	beta-Alanine	39.4±7.5	33.7±9.1	0.434	64.4±15.6	81.5±24.7	0.35
**biogenic amines**	gamma-Aminobutyric acid	72.9±11.7	68.4±8	0.597	82.8±11.8	88.5±32.1	0.782
**biogenic amines**	Histamine	3.6±1.3	3.4±1.9	0.877	0.6±0.2	1.3±0.9	0.219

**biogenic amines**	Putrescine	6.3±1.9	6.2±1.6	0.977	7.6±2.1	10.9±3.5	0.205
**biogenic amines**	Serotonin	2.4±1.3	2.4±1.2	0.985	1.7±0.7	2.6±1.7	0.379
**biogenic amines**	Spermidine	9.2±2.5	9.3±2.2	0.991	16.5±7.5	18.9±9.9	0.753
**biogenic amines**	Spermine	5.5±1.2	7.1±2.6	0.353	9.3±4.6	8.8±4.1	0.882
**Carboxylic acids**	cis-Aconitic acid	1.1±0.6	0.8±0.3	0.381	1.2±0.5	3.2±2.6	0.236
**Carboxylic acids**	Dodecanedioic acid	1.1±0.3	1.1±0.2	0.999	1.3±0.3	1.3±0.2	0.852
**Carboxylic acids**	Hippuric acid	7.6±6.4	3±1.4	0.264	8.5±4.8	18.6±12.2	0.231
**Carboxylic acids**	L-Lactic acid	7244.3±1189.4	7813.8±2648.2	0.746	16340.8±3331.1	18165.8±3955.6	0.564
**Carboxylic acids**	3-Hydroxyglutaric acid	20.1±1.3	17.1±4.3	0.284	19.3±5.6	16.5±1.5	0.42
**Carboxylic acids**	Succinic acid	81.7±13.9	112.3±48.9	0.336	190.1±70	207.5±61.5	0.757
**Ceramides**	Cer(d16:1/22:0)	0.1±0.1	0.1±0.1	0.894	0.1±0.1	0.1±0.1	0.376
**Ceramides**	Cer(d16:1/24:0)	0.2±0.1	0.2±0.1	0.238	0.1±0.1	0.1±0.1	0.89
**Ceramides**	Cer(d18:1/14:0)	0.2±0.1	0.2±0.1	0.744	0.1±0.1	0.1±0.1	0.413
**Ceramides**	Cer(d18:1/16:0)	4.7±0.4	5±0.7	0.493	5.2±0.7	5.7±0.9	0.504
**Ceramides**	Cer(d18:1/18:0(OH))	0.4±0.2	0.4±0.1	0.841	0.6±0.2	0.6±0.1	0.651
**Ceramides**	Cer(d18:1/18:0)	0.9±0.1	0.9±0.1	0.622	1.1±0.2	1.1±0.1	0.952
**Ceramides**	Cer(d18:1/18:1)	0.2±0.1	0.1±0.1	0.095	0.2±0.1	0.2±0.1	0.549
**Ceramides**	Cer(d18:1/20:0(OH))	26.4±3.4	24.4±2.5	0.426	36±6	38±6.1	0.698
**Ceramides**	Cer(d18:1/20:0)	1±0.1	1±0.2	0.946	1.3±0.3	1.5±0.1	0.349
**Ceramides**	Cer(d18:1/22:0)	7.6±0.2	7.9±0.9	0.684	8.9±1.6	9.6±0.5	0.474
**Ceramides**	Cer(d18:1/23:0)	3.2±0.2	2.9±0.6	0.383	3.7±0.9	3.8±0.8	0.937
**Ceramides**	Cer(d18:1/24:0)	18.5±1.2	18±2.1	0.688	16.8±1.1	18±2.1	0.39
**Ceramides**	Cer(d18:1/24:1)	9.5±0.4	9.1±0.4	0.193	9.9±1.1	10.2±1.3	0.782
**Ceramides**	Cer(d18:1/25:0)	1.3±0.1	0.9±0.2	0.002*	1.5±0.1	1.5±0.2	0.845
**Ceramides**	Cer(d18:1/26:0)	0.3±0.1	0.2±0.1	0.101	0.2±0.1	0.2±0.2	0.23
**Ceramides**	Cer(d18:1/26:1)	0.2±0.1	0.2±0.1	0.553	0.2±0.1	0.3±0.1	0.111
**Ceramides**	Cer(d18:2/14:0)	0.1±0.1	0.1±0	0.356	0.1±0.1	0.1±0	0.356
**Ceramides**	Cer(d18:2/16:0)	3.1±0.4	3.4±0.6	0.509	3.3±0.5	4.2±0.6	0.088
**Ceramides**	Cer(d18:2/18:0)	0.3±0.1	0.4±0.1	0.36	0.3±0.1	0.3±0.1	0.922
**Ceramides**	Cer(d18:2/20:0)	0.3±0.1	0.4±0.1	0.477	0.4±0.1	0.4±0.1	0.878
**Ceramides**	Cer(d18:2/22:0)	6.1±1	7±0.7	0.208	7±1	7.9±0.9	0.283
**Ceramides**	Cer(d18:2/23:0)	0.9±0.2	0.9±0.3	0.966	1±0.2	1.1±0.3	0.655
**Ceramides**	Cer(d18:2/24:0)	22.2±3.3	21.4±1.4	0.704	20.9±1.8	22.8±2.8	0.362
**Ceramides**	Cer(d18:2/24:1)	5.4±0.9	5.8±0.5	0.418	6.5±0.9	6.6±0.5	0.857
**Cholesteryl esters**	CE(14:0)	0.3±0.2	0.3±0.4	0.718	0.7±0.5	0.7±0.7	0.912
**Cholesteryl esters**	CE(15:1)	2±0.7	1.6±1.6	0.684	2.3±1.7	3.5±1.5	0.392
**Cholesteryl esters**	CE(16:1)	1.1±0.3	1.5±1	0.539	1.1±0.3	1.4±0.7	0.444
**Cholesteryl esters**	CE(18:1)	2.8±0.9	3.5±1.9	0.597	3.1±0.8	5±1.3	0.075
**Cholesteryl esters**	CE(18:2)	9.9±4.6	16.8±11.9	0.38	15.6±2.2	23.5±11.8	0.293

**Cholesteryl esters**	CE(18:3)	0.9±0.6	0.9±0.6	0.888	0.6±0.3	1±0.6	0.289
**Cholesteryl esters**	CE(20:3)	0.5±0.4	0.9±0.5	0.267	1.1±0.5	1.6±0.9	0.367
**Cholesteryl esters**	CE(20:4)	19.2±4.8	31.1±19.4	0.342	20.9±2.6	29.3±10.8	0.236
**Cholesteryl esters**	CE(20:5)	0.6±0.3	0.8±0.5	0.482	0.7±0.3	1.3±0.6	0.15
**Cholesteryl esters**	CE(22:5)	0.5±0.2	0.6±0.3	0.45	0.6±0.1	0.8±0.2	0.083
**Cholesteryl esters**	CE(22:6)	5.4±1.5	6.8±3.6	0.551	3.7±0.3	4.6±1.9	0.423
**Diacylglyceroles**	DG(14:0_18:1)	0.9±0.4	1.2±0.5	0.353	0.8±0.3	1.2±0.2	0.06
**Diacylglyceroles**	DG(14:0_18:2)	0.8±0.3	1.1±0.5	0.379	0.9±0.1	1.4±0.4	0.065
**Diacylglyceroles**	DG(14:1_18:1)	0.2±0.2	0.3±0.1	0.215	0.3±0.2	0.3±0.1	0.717
**Diacylglyceroles**	DG(16:0_16:1)	2.8±1.2	4.8±2.1	0.191	3.8±1.5	6.3±0.7	0.033*§
**Diacylglyceroles**	DG(16:0_18:1)	21.5±5	27.7±8.4	0.313	26.3±7.4	39.5±3.6	0.032*§
**Diacylglyceroles**	DG(16:0_18:2)	16.2±4.7	19.6±6.7	0.493	23.3±4.6	32±5.1	0.068
**Diacylglyceroles**	DG(16:0_20:3)	0.4±0.2	0.5±0.1	0.272	0.4±0.1	0.6±0.3	0.196
**Diacylglyceroles**	DG(16:0_20:4)	1.3±0.3	1.6±0.4	0.372	1.5±0.4	1.9±0.5	0.267
**Diacylglyceroles**	DG(16:1_18:0)	0.3±0.2	0.2±0.2	0.822	0.4±0.2	0.5±0.2	0.362
**Diacylglyceroles**	DG(16:1_18:2)	1.8±0.8	2.7±1.3	0.316	2.6±0.5	4.8±0.5	0.002*§
**Diacylglyceroles**	DG(17:0_18:1)	6.2±1.1	4.7±0.5	0.059	6.8±1.4	6.8±1.9	0.967
**Diacylglyceroles**	DG(18:0_20:4)	5.7±0.3	5.5±1.3	0.795	5.9±0.6	6.5±1.6	0.514
**Diacylglyceroles**	DG(18:1_18:1)	6.7±2.7	9.3±3.6	0.34	8±1.1	12.9±1.4	0.003*§
**Diacylglyceroles**	DG(18:1_18:2)	17.2±5.9	23±9.6	0.404	29.7±4.3	44.6±12.4	0.096
**Diacylglyceroles**	DG(18:1_18:3)	1.6±0.5	1.5±1.3	0.854	2.2±0.8	1.8±0.7	0.563
**Diacylglyceroles**	DG(18:1_20:0)	0.8±0.4	0.7±0.4	0.729	0.4±0.3	1.1±0.4	0.03*§
**Diacylglyceroles**	DG(18:1_20:1)	0.2±0.1	0.3±0.1	0.23	0.3±0.1	0.5±0.1	0.069
**Diacylglyceroles**	DG(18:1_20:2)	0.3±0.1	0.2±0.1	0.556	0.3±0.1	0.5±0.2	0.265
**Diacylglyceroles**	DG(18:1_20:4)	1.8±0.3	1.9±0.3	0.679	1.9±0.4	2.5±0.9	0.31
**Diacylglyceroles**	DG(18:2_18:2)	12.2±2.5	14.8±7	0.565	26.4±4.3	35.7±13.1	0.285
**Diacylglyceroles**	DG(18:2_20:4)	0.7±0.1	0.9±0.3	0.309	1.1±0.1	1.5±0.6	0.378
**Dihydroceramides**	Cer(d18:0/18:0(OH))	5.1±2.1	4.5±4	0.806	4.6±2.4	6.1±3.4	0.543
**Dihydroceramides**	Cer(d18:0/22:0)	0.5±0.1	0.5±0.1	0.795	0.5±0.2	0.6±0.3	0.54
**Dihydroceramides**	Cer(d18:0/24:0)	0.8±0.2	0.7±0.1	0.318	0.6±0.1	0.7±0.3	0.264
**Dihydroceramides**	Cer(d18:0/24:1)	0.5±0.1	0.5±0.2	0.484	0.5±0.1	0.6±0.2	0.532
**Fatty acids**	Arachidonic acid	483.3±289.5	318±68.2	0.374	292.5±56	268.8±77.2	0.681
**Fatty acids**	Docosahexaenoic acid	242.3±114.9	155±43	0.264	107.2±24.9	94.7±35.7	0.637
**Fatty acids**	Eicosapentaenoic acid	4.5±3.1	5±0.9	0.796	3.4±0.5	3.5±0.9	0.85
**Fatty acids**	Elaidic acid	73.1±16.6	101.1±15.9	0.079	121.3±24.3	112.6±17.5	0.63
**Fatty acids**	Linoleic acid	241±83.9	223.5±17.9	0.736	304.3±96	289.3±92.9	0.853
**Fatty acids**	Eicosadienoic acid	2.8±1	3±0.4	0.732	5±1.1	4.2±1	0.368
**Fatty acids**	Dihomo-gamma-linolenic acid	10.5±3.6	11.8±1.4	0.561	10.5±3.7	13.5±3.9	0.367
**Lysophosphatidylcholines**	lysoPC a C14:0	1.1±0.2	1.1±0.2	0.781	1.1±0.2	1.3±0.3	0.143

**Lysophosphatidylcholines**	lysoPC a C16:0	203.5±8.1	184.8±35.1	0.402	181.8±13.3	199±26.5	0.352
**Lysophosphatidylcholines**	lysoPC a C16:1	2.8±0.6	3.1±0.7	0.55	3.1±0.8	4.1±1.1	0.198
**Lysophosphatidylcholines**	lysoPC a C17:0	3.4±0.4	2.6±0.6	0.066	4±0.4	3.9±1	0.991
**Lysophosphatidylcholines**	lysoPC a C18:0	104.5±4.1	96.8±24.5	0.607	92.7±12.1	91.9±14.8	0.947
**Lysophosphatidylcholines**	lysoPC a C18:1	39.8±3.8	38.6±10.9	0.869	50.8±10.4	58.3±15.6	0.514
**Lysophosphatidylcholines**	lysoPC a C18:2	43.1±6.3	32.4±12	0.216	69.3±16.1	71.7±30.1	0.908
**Lysophosphatidylcholines**	lysoPC a C20:3	4.4±1.6	4.7±1.8	0.844	8.2±3.1	9.1±4.5	0.792
**Lysophosphatidylcholines**	lysoPC a C20:4	64.9±15.9	66.6±23	0.922	83.2±19	81.4±39.1	0.945
**Lysophosphatidylcholines**	lysoPC a C24:0	1.8±0.2	2±0.3	0.551	2±0.2	2.2±0.4	0.347
**Lysophosphatidylcholines**	lysoPC a C26:0	1.5±0.4	1.7±0.5	0.541	1.3±0.4	1.6±0.4	0.248
**Lysophosphatidylcholines**	lysoPC a C26:1	0.8±0.1	0.8±0.2	0.958	0.8±0.2	0.7±0.2	0.251
**Lysophosphatidylcholines**	lysoPC a C28:0	1.3±0.3	2±0.8	0.162	1.5±0.8	1.9±1.1	0.57
**Lysophosphatidylcholines**	lysoPC a C28:1	1.5±0.7	1.8±0.8	0.595	1.5±0.3	2.4±1.4	0.336
**Phosphatidylcholines**	PC aa C24:0	0.4±0.1	0.5±0.1	0.06	0.6±0.1	0.5±0.1	0.204
**Phosphatidylcholines**	PC aa C26:0	2.1±0.2	2.4±0.4	0.214	2.4±0.5	2.5±0.2	0.801
**Phosphatidylcholines**	PC aa C28:1	2.2±0.1	2.4±0.2	0.089	2±0.3	2.4±0.6	0.266
**Phosphatidylcholines**	PC aa C30:0	39.3±5.4	44±6.4	0.365	37.4±3.2	43.5±5.2	0.132
**Phosphatidylcholines**	PC aa C30:2	0.2±0.1	0.3±0.3	0.505	0.1±0.1	0.1±0.2	0.266
**Phosphatidylcholines**	PC aa C32:0	2297.3±202.5	2381.3±399.2	0.757	2034.5±126	2043.3±380.1	0.972
**Phosphatidylcholines**	PC aa C32:1	167.5±19.7	214±31.9	0.075	178.5±13.8	214.3±27.6	0.092
**Phosphatidylcholines**	PC aa C32:2	12.4±2	13.7±2.4	0.493	15±1.9	17.2±1.6	0.178
**Phosphatidylcholines**	PC aa C32:3	2.1±0.3	2.5±0.6	0.302	2.8±0.3	2.9±0.5	0.793
**Phosphatidylcholines**	PC aa C34:1	1618.5±85.6	1634±250.1	0.923	1715.3±103.6	1948±153.6	0.073
**Phosphatidylcholines**	PC aa C34:2	1362±102.1	1175±176.3	0.163	1886.3±251.7	2034±101.5	0.382
**Phosphatidylcholines**	PC aa C34:3	58±7.4	56.1±8.8	0.781	78.4±8.4	83.6±7.1	0.442
**Phosphatidylcholines**	PC aa C34:4	7.6±0.9	7.5±1.4	0.946	10.4±1.6	10.9±0.9	0.615
**Phosphatidylcholines**	PC aa C36:0	52.8±5	43.1±4	0.038*	46.9±8.4	43.5±4.8	0.558
**Phosphatidylcholines**	PC aa C36:1	480.3±12.3	442.8±77.9	0.442	478.5±48.2	523.3±65.1	0.375
**Phosphatidylcholines**	PC aa C36:2	909.8±29.6	743.3±130.2	0.074	1059.3±116.6	1117.5±85.2	0.511
**Phosphatidylcholines**	PC aa C36:3	475.5±31.8	421.5±41.8	0.125	664.5±76.4	721.5±75.7	0.394
**Phosphatidylcholines**	PC aa C36:4	2349.8±170.6	2315±456	0.906	2349.8±99.3	2353.8±237.3	0.98
**Phosphatidylcholines**	PC aa C36:5	47±1.9	52.1±10.5	0.442	51.8±3.1	58±8.7	0.288
**Phosphatidylcholines**	PC aa C36:6	24±2.1	20.3±5	0.271	22.2±3.4	22.3±5.4	0.99
**Phosphatidylcholines**	PC aa C38:0	409.3±27.3	292.8±21.7	0.002*	284.8±100.8	259.3±102.8	0.77
**Phosphatidylcholines**	PC aa C38:1	125.8±4.8	89±13	0.004*	109.4±33.1	91.5±29.7	0.513
**Phosphatidylcholines**	PC aa C38:3	158.5±12.1	141.3±13.9	0.155	182.3±26	194.3±29.6	0.616
**Phosphatidylcholines**	PC aa C38:4	1416±54.1	1312.8±223.4	0.467	1423.5±168.6	1416±199.2	0.962
**Phosphatidylcholines**	PC aa C38:5	627.3±28.5	610.5±67.6	0.706	681.8±64.5	741±95.1	0.407
**Phosphatidylcholines**	PC aa C38:6	2271.8±156.9	2152.8±487.8	0.702	1629.8±72.9	1664±467.6	0.905

**Phosphatidylcholines**	PC aa C40:1	3.8±0.5	3±0.1	0.029*	4.4±1.2	4.1±0.6	0.775
**Phosphatidylcholines**	PC aa C40:2	5.6±1.3	4.4±0.3	0.138	6±1.3	5.9±0.8	0.884
**Phosphatidylcholines**	PC aa C40:3	5.8±1.1	4.7±0.2	0.109	6.8±1.1	6.8±0.6	0.979
**Phosphatidylcholines**	PC aa C40:4	22.7±1.4	23.1±4.1	0.885	27.6±2.8	30.2±3.4	0.333
**Phosphatidylcholines**	PC aa C40:5	55.4±2.2	57.7±6	0.545	65.2±6.6	76.9±7.1	0.081
**Phosphatidylcholines**	PC aa C40:6	400.3±18.8	331±56.8	0.092	292.8±27.9	296±70.3	0.944
**Phosphatidylcholines**	PC aa C42:0	1.3±0.3	1±0.2	0.065	1±0.2	1±0.2	0.754
**Phosphatidylcholines**	PC aa C42:1	1.9±0.5	1.4±0.3	0.111	1.4±0.2	1.4±0.4	0.801
**Phosphatidylcholines**	PC aa C42:2	2.2±0.7	1.5±0.2	0.107	2±0.3	1.9±0.4	0.756
**Phosphatidylcholines**	PC aa C42:4	7.6±1.1	5.5±1.3	0.066	3.7±0.7	3.9±0.9	0.797
**Phosphatidylcholines**	PC aa C42:5	9±0.5	7.8±0.4	0.013*	7.5±1	7.3±1.2	0.798
**Phosphatidylcholines**	PC aa C42:6	5.4±1.3	4.1±0.5	0.123	5.4±0.5	5.6±0.6	0.617
**Phosphatidylcholines**	PC ae C30:0	4.6±0.6	4.7±0.6	0.881	4.3±1	5±0.8	0.361
**Phosphatidylcholines**	PC ae C30:1	0.4±0.2	0.5±0.2	0.282	0.4±0.1	0.5±0.2	0.463
**Phosphatidylcholines**	PC ae C30:2	0.6±0.2	0.5±0.1	0.459	0.6±0.1	0.7±0.2	0.516
**Phosphatidylcholines**	PC ae C32:1	31.5±2.9	32.1±4.1	0.829	27.6±5.7	30.4±4.5	0.527
**Phosphatidylcholines**	PC ae C32:2	4.4±0.2	4.5±1	0.818	5±1.2	5.3±0.5	0.742
**Phosphatidylcholines**	PC ae C34:0	32±4.2	28.6±7.4	0.506	43.8±7.3	42.9±9.2	0.901
**Phosphatidylcholines**	PC ae C34:1	203.8±12.2	170±20.3	0.049*	186.8±33.2	186±23.5	0.976
**Phosphatidylcholines**	PC ae C34:2	77±3.3	65.5±12.4	0.171	88±18.2	93±7.4	0.677
**Phosphatidylcholines**	PC ae C34:3	14.7±0.7	12±2.1	0.071	18.1±4.7	20.2±2.1	0.505
**Phosphatidylcholines**	PC ae C36:0	7.5±0.5	8.6±2.8	0.539	10.3±2.3	12.5±1.8	0.215
**Phosphatidylcholines**	PC ae C36:1	33.1±1.9	30.2±8	0.566	47.2±9.3	53.9±8.9	0.398
**Phosphatidylcholines**	PC ae C36:2	34±1.6	26.1±4.9	0.035*	51.2±12.8	54.6±8.8	0.717
**Phosphatidylcholines**	PC ae C36:3	31.1±1.1	22.9±3.1	0.005*	35.9±8.5	36.9±5.5	0.869
**Phosphatidylcholines**	PC ae C36:4	201±6.8	180±32.4	0.313	223±50.7	227.8±27.1	0.891
**Phosphatidylcholines**	PC ae C36:5	160.3±8.2	155±17.9	0.66	166±26.5	177.8±17	0.541
**Phosphatidylcholines**	PC ae C38:0	50.9±5.4	46.9±7.3	0.472	39.8±5.6	40.6±11.1	0.909
**Phosphatidylcholines**	PC ae C38:1	5.3±2	3±0.8	0.101	4.8±1.7	6.2±1.7	0.32
**Phosphatidylcholines**	PC ae C38:2	8.9±0.4	6.5±1.5	0.038*	12.3±3	14.1±1.8	0.409
**Phosphatidylcholines**	PC ae C38:3	9.3±0.5	7.9±1.2	0.114	13.1±3.3	13.9±1.6	0.721
**Phosphatidylcholines**	PC ae C38:4	55.6±4.2	50.1±11.7	0.471	81±11.2	83.9±10.3	0.755
**Phosphatidylcholines**	PC ae C38:5	130.5±3.3	119.5±9.7	0.111	178.5±27.8	190.3±18.1	0.562
**Phosphatidylcholines**	PC ae C38:6	1013±102.4	811±142	0.093	703.3±108	695.5±99.1	0.931
**Phosphatidylcholines**	PC ae C40:1	101.1±5	89.5±13.2	0.202	99.9±6	90.9±17.4	0.43
**Phosphatidylcholines**	PC ae C40:2	4.6±0.2	4.5±0.7	0.852	5.4±0.9	5.4±1.4	0.994
**Phosphatidylcholines**	PC ae C40:3	4.8±1.1	4.9±2.3	0.94	5.1±0.9	4.8±0.8	0.614
**Phosphatidylcholines**	PC ae C40:4	12.8±1	12.2±2.9	0.72	16.6±2.8	15.9±3.9	0.818
**Phosphatidylcholines**	PC ae C40:5	12.2±1.3	12.2±2.4	0.975	15.5±2.6	16.8±1.1	0.434

**Phosphatidylcholines**	PC ae C40:6	71.3±4.2	51.2±8.9	0.012*	69.7±10.7	72.7±14.5	0.782
**Phosphatidylcholines**	PC ae C42:0	6.6±0.5	5.6±0.3	0.012*	6.8±1.1	6.9±0.8	0.993
**Phosphatidylcholines**	PC ae C42:1	8.3±0.7	7.5±1.2	0.311	9.9±1.5	10.6±1.1	0.53
**Phosphatidylcholines**	PC ae C42:2	5.7±0.4	6.1±0.7	0.417	5.8±0.4	6.3±0.6	0.191
**Phosphatidylcholines**	PC ae C42:3	24.6±1.2	27±4.7	0.419	15.8±1.8	16.3±4.7	0.874
**Phosphatidylcholines**	PC ae C42:4	1.6±0.2	1.5±0.3	0.647	1.6±0.4	1.7±0.3	0.688
**Phosphatidylcholines**	PC ae C42:5	3.9±0.3	3.7±0.6	0.429	3.8±0.4	3.8±0.3	0.899
**Phosphatidylcholines**	PC ae C44:3	1.7±0.2	1.4±0.2	0.096	1.6±0.3	1.5±0.4	0.855
**Phosphatidylcholines**	PC ae C44:4	1.5±0.2	1.3±0.1	0.053	1.3±0.2	1.3±0.4	0.819
**Phosphatidylcholines**	PC ae C44:5	2.4±0.1	2±0.4	0.131	1.6±0.3	1.6±0.4	0.875
**Phosphatidylcholines**	PC ae C44:6	1±0.2	1±0.1	0.848	1±0.4	1±0.2	0.991
**Dihexosylceramides**	Hex2Cer(d18:1/14:0)	2±0.4	1.8±0.5	0.547	2±0.6	2.3±0.7	0.548
**Dihexosylceramides**	Hex2Cer(d18:1/16:0)	3.6±0.6	3.1±0.7	0.327	3.9±0.9	4±0.9	0.869
**Dihexosylceramides**	Hex2Cer(d18:1/18:0)	3.2±0.6	2.6±0.6	0.289	2.8±0.6	2.9±0.7	0.765
**Dihexosylceramides**	Hex2Cer(d18:1/20:0)	0.3±0.1	0.3±0.1	0.549	0.4±0.1	0.5±0.1	0.553
**Dihexosylceramides**	Hex2Cer(d18:1/22:0)	0.8±0.2	0.8±0.3	0.945	0.8±0.1	0.9±0.1	0.112
**Dihexosylceramides**	Hex2Cer(d18:1/24:0)	0.7±0.1	0.7±0.2	0.735	0.8±0.1	0.8±0.1	0.24
**Dihexosylceramides**	Hex2Cer(d18:1/24:1)	0.4±0.1	0.4±0.1	0.94	0.5±0.1	0.6±0.1	0.444
**Dihexosylceramides**	Hex2Cer(d18:1/26:0)	0.1±0.1	0.1±0.1	0.067	0.1±0.1	0.1±0.1	0.999
**Dihexosylceramides**	Hex2Cer(d18:1/26:1)	0.3±0.1	0.4±0.2	0.362	0.4±0.1	0.4±0.1	0.492
**Trihexosylceramides**	Hex3Cer(d18:1/16:0)	1.7±0.2	1.6±0.2	0.289	4.2±0.7	4.3±1.4	0.898
**Trihexosylceramides**	Hex3Cer(d18:1/18:0)	0.6±0.2	0.6±0.1	0.829	1±0.3	1.1±0.2	0.529
**Trihexosylceramides**	Hex3Cer(d18:1/24:1)	1.2±0.1	1.1±0.2	0.092	2.8±0.6	2.8±0.6	0.926
**Trihexosylceramides**	Hex3Cer(d18:1/26:1)	1.7±0.2	1.9±0.3	0.452	1.7±0.9	1.8±0.4	0.748
**Trihexosylceramides**	Hex3Cer(d18:1_20:0)	1.3±0.2	1.1±0.2	0.161	3.6±1.4	3.5±0.7	0.847
**Trihexosylceramides**	Hex3Cer(d18:1_22:0)	2.9±0.3	2.7±0.5	0.566	6.5±2	6.4±1.5	0.934
**Hexosylceramides**	HexCer(d16:1/22:0)	0.1±0.1	0.1±0.1	0.128	0.1±0.1	0.1±0.1	0.78
**Hexosylceramides**	HexCer(d18:1/14:0)	0.1±0.1	0.1±0.1	0.253	0.1±0.1	0.1±0.1	0.306
**Hexosylceramides**	HexCer(d18:1/16:0)	3.3±0.5	3.3±0.5	0.938	5.4±1	5.7±0.5	0.617
**Hexosylceramides**	HexCer(d18:1/18:0)	0.8±0.1	0.9±0.2	0.264	1.4±0.5	1.3±0.2	0.796
**Hexosylceramides**	HexCer(d18:1/18:1)	0.4±0.1	0.4±0.1	0.166	0.5±0.2	0.5±0.1	0.706
**Hexosylceramides**	HexCer(d18:1/20:0)	2.8±0.5	2.7±0.5	0.626	5.5±2	5.2±0.6	0.83
**Hexosylceramides**	HexCer(d18:1/22:0)	14.6±1.8	12.6±2.6	0.295	20.6±4.6	22±2.2	0.649
**Hexosylceramides**	HexCer(d18:1/23:0)	3.6±0.5	3±0.5	0.237	4.9±0.9	5.2±1.2	0.752
**Hexosylceramides**	HexCer(d18:1/24:0)	7.4±0.3	6.3±0.7	0.036*	8.9±1.7	8.9±0.5	0.981
**Hexosylceramides**	HexCer(d18:1/24:1)	15.5±2.5	13.7±1.8	0.366	17.8±2.3	19.7±2.8	0.386
**Hexosylceramides**	HexCer(d18:1/26:0)	0.3±0.1	0.3±0.1	0.33	0.3±0.1	0.4±0.1	0.715
**Hexosylceramides**	HexCer(d18:1/26:1)	0.9±0.2	0.9±0.3	0.804	1±0.2	1.2±0.2	0.159
**Hexosylceramides**	HexCer(d18:2/16:0)	0.2±0.1	0.2±0.1	0.774	0.2±0.1	0.3±0.1	0.43

**Hexosylceramides**	HexCer(d18:2/20:0)	0.1±0.1	0.1±0.1	0.59	0.2±0.1	0.2±0.1	0.801
**Hexosylceramides**	HexCer(d18:2/22:0)	1.6±0.2	1.3±0.1	0.038*	2.4±0.6	2.1±0.5	0.545
**Hexosylceramides**	HexCer(d18:2/23:0)	0.8±0.2	0.6±0.2	0.097	1±0.2	0.8±0.2	0.448
**Hexosylceramides**	HexCer(d18:2/24:0)	4.4±0.4	3.4±0.2	0.009*	5.8±1.1	5.4±1.2	0.662
**Indoles and derivatives**	Indoleacetic acid	2±0.7	1.3±0.2	0.123	4.4±3	6.6±3.5	0.429
**Indoles and derivatives**	Indole-3-propionic acid	0.9±0.4	0.7±0.3	0.461	2.3±1.5	1.8±0.8	0.635
**Indoles and derivatives**	Indoxyl sulfate	1.8±0.5	1.8±1	0.985	3.1±1.7	5.8±3	0.216
**Sphingomyelins**	SM (OH) C14:1	21±1.5	18.9±3.4	0.36	20.9±2.7	20±4.7	0.781
**Sphingomyelins**	SM (OH) C16:1	9.4±0.4	7.9±1.4	0.122	13±3.3	13.4±3.9	0.904
**Sphingomyelins**	SM (OH) C22:1	49.8±2.7	39.9±8.7	0.107	53.1±8.1	54±14.6	0.925
**Sphingomyelins**	SM (OH) C22:2	16.7±0.5	13.8±4.3	0.283	16±2.8	16.9±5.6	0.813
**Sphingomyelins**	SM (OH) C24:1	5.6±1.1	4.8±1	0.385	5.1±0.6	5.5±1.2	0.555
**Sphingomyelins**	SM C16:0	1223.3±110	1117.5±115.5	0.295	1092.3±103.4	1133±211.1	0.775
**Sphingomyelins**	SM C16:1	221.5±19.5	199±12.6	0.143	204±11.9	213±30.4	0.649
**Sphingomyelins**	SM C18:0	60.9±5.3	58.2±6.5	0.591	56.9±7.8	59.7±5.9	0.632
**Sphingomyelins**	SM C18:1	10±1.1	9.7±1.5	0.775	8.6±0.9	9.3±1	0.331
**Sphingomyelins**	SM C20:2	0.2±0.2	0.2±0.2	0.962	0.2±0.2	0.2±0.3	0.884
**Sphingomyelins**	SM C22:3	3.9±0.8	3.2±2.3	0.635	3.7±2.5	3.5±2	0.952
**Sphingomyelins**	SM C24:0	232.3±6.1	204.8±26.5	0.131	214.5±28.6	224±40.2	0.75
**Sphingomyelins**	SM C24:1	343.5±17.3	309.5±32.4	0.16	305±30.6	319±50.2	0.695
**Sphingomyelins**	SM C26:0	1.3±0.2	1.3±0.5	0.98	1.3±0.2	2±0.5	0.038*§
**Sphingomyelins**	SM C26:1	1.7±0.5	1.3±0.1	0.134	1.5±0.3	1.6±0.5	0.873
**Triacylglycerole**	TG(14:0_32:2)	2.7±2.4	2.8±2.3	0.991	0.8±0.5	5.5±7.7	0.331
**Triacylglycerole**	TG(14:0_34:0)	3.8±3.7	4.5±4.7	0.847	1.1±0.4	7.1±9.7	0.32
**Triacylglycerole**	TG(14:0_34:1)	23.9±23.1	26.9±29.7	0.894	4.5±1.2	48.8±70.7	0.32
**Triacylglycerole**	TG(14:0_34:2)	21±18.6	22.5±26	0.938	4.2±1.6	43±58.4	0.295
**Triacylglycerole**	TG(14:0_34:3)	3.4±3.3	3.8±4.1	0.899	0.8±0.3	8.3±11.5	0.303
**Triacylglycerole**	TG(14:0_35:1)	0.5±0.3	0.5±0.3	0.902	0.2±0.2	1±1	0.209
**Triacylglycerole**	TG(14:0_35:2)	0.5±0.5	0.4±0.4	0.766	0.3±0.1	1±1.1	0.308
**Triacylglycerole**	TG(14:0_36:1)	7.4±6.6	6.8±8.4	0.927	0.8±0.2	10.1±15.1	0.33
**Triacylglycerole**	TG(14:0_36:2)	24.1±21.9	21.4±25.5	0.895	2.6±0.5	34.8±51.7	0.322
**Triacylglycerole**	TG(14:0_36:3)	20.4±18.2	16.7±21.1	0.825	3.3±0.9	30.1±42.2	0.313
**Triacylglycerole**	TG(14:0_36:4)	5.1±3.9	4±4.4	0.766	1.5±0.4	7.9±9	0.26
**Triacylglycerole**	TG(14:0_38:4)	0.8±0.2	0.9±0.5	0.788	0.5±0.2	1.2±0.6	0.081
**Triacylglycerole**	TG(14:0_38:5)	0.5±0.2	0.7±0.4	0.428	0.3±0.2	0.7±0.4	0.102
**Triacylglycerole**	TG(16:0_28:1)	1.7±0.9	2.1±0.9	0.596	0.5±0.2	3.6±4.1	0.229
**Triacylglycerole**	TG(16:0_28:2)	0.8±0.5	0.9±0.5	0.833	0.5±0.2	2.1±2.1	0.236
**Triacylglycerole**	TG(16:0_30:2)	2.1±2.1	3.6±2	0.376	0.7±0.5	5.1±6.1	0.262
**Triacylglycerole**	TG(16:0_32:0)	32.9±34.2	40.2±38.2	0.813	10.5±5.7	67.3±74.4	0.235

**Triacylglycerole**	TG(16:0_32:1)	51.7±50.6	60.2±64.9	0.863	10.6±3.9	154±228.9	0.32
**Triacylglycerole**	TG(16:0_32:2)	22.3±20.6	25.2±28.9	0.89	5.9±2.1	56.6±78.9	0.308
**Triacylglycerole**	TG(16:0_32:3)	1.5±1.3	1.8±1.7	0.797	0.7±0.3	3.7±4.2	0.251
**Triacylglycerole**	TG(16:0_33:1)	2.7±2.2	3.4±2.2	0.733	1.6±0.8	8±7.1	0.169
**Triacylglycerole**	TG(16:0_33:2)	1.7±1	1.7±1.5	0.955	1.4±0.4	4.9±4.4	0.219
**Triacylglycerole**	TG(16:0_34:0)	21.4±21.8	25.6±27.2	0.84	7±1.8	47.7±60.4	0.288
**Triacylglycerole**	TG(16:0_34:1)	207.6±187.6	227.7±246.1	0.914	55.7±17.2	525.7±714.4	0.299
**Triacylglycerole**	TG(16:0_34:2)	186.2±166.6	191.7±222	0.974	62.1±19.7	465.6±598	0.287
**Triacylglycerole**	TG(16:0_34:3)	37.3±31.3	35.3±44	0.953	9±4.4	97±133.1	0.296
**Triacylglycerole**	TG(16:0_34:4)	1.4±1	2.1±1.8	0.591	1.1±0.4	4.3±4.3	0.246
**Triacylglycerole**	TG(16:0_35:1)	2.6±2.2	2.8±2.5	0.901	1.1±0.4	8.9±11.7	0.294
**Triacylglycerole**	TG(16:0_35:2)	3.4±2.7	3.4±3.4	0.986	1.8±0.3	9.1±10.5	0.271
**Triacylglycerole**	TG(16:0_35:3)	1.6±1.4	1.4±1.6	0.908	0.9±0.3	3.9±3.5	0.198
**Triacylglycerole**	TG(16:0_36:2)	236.1±226.3	201.8±246.6	0.865	37.8±8.4	391.1±549	0.308
**Triacylglycerole**	TG(16:0_36:3)	211.3±190.5	167.3±215	0.8	53.5±11.3	379.3±490.3	0.294
**Triacylglycerole**	TG(16:0_36:4)	52.9±42.7	43±51.3	0.807	25.8±6.7	108.9±117.9	0.269
**Triacylglycerole**	TG(16:0_36:5)	3.2±2.1	3.1±3.3	0.989	2±0.5	7.3±7.3	0.251
**Triacylglycerole**	TG(16:0_36:6)	0.7±0.1	0.9±0.6	0.417	0.5±0.1	1±0.6	0.163
**Triacylglycerole**	TG(16:0_37:3)	1±0.8	0.9±1	0.831	0.6±0.1	2.9±3.6	0.295
**Triacylglycerole**	TG(16:0_38:1)	1.7±1.4	2.1±2.5	0.778	0.7±0.1	3.7±4.4	0.277
**Triacylglycerole**	TG(16:0_38:2)	6.7±6.1	5.9±6.9	0.879	2.2±0.5	11±13.9	0.315
**Triacylglycerole**	TG(16:0_38:3)	6.6±5.4	5.6±6.2	0.831	2.9±0.5	12.1±13.5	0.281
**Triacylglycerole**	TG(16:0_38:4)	4.3±2.8	5.2±3.9	0.753	3.9±0.8	9.3±5.9	0.166
**Triacylglycerole**	TG(16:0_38:5)	4.7±2.4	6±3.7	0.624	4.3±0.8	8.7±4.7	0.15
**Triacylglycerole**	TG(16:0_38:6)	5±1.5	7±3.8	0.42	4.4±1.4	6.5±2.3	0.221
**Triacylglycerole**	TG(16:0_38:7)	0.7±0.5	1±0.5	0.573	0.6±0.3	1.9±1.9	0.28
**Triacylglycerole**	TG(16:0_40:6)	1.6±0.8	1.8±1.1	0.727	1.6±0.5	3.2±0.9	0.027*§
**Triacylglycerole**	TG(16:0_40:7)	1.9±0.8	2.4±1.5	0.586	2.2±0.9	3.4±1.4	0.246
**Triacylglycerole**	TG(16:0_40:8)	1.6±0.6	1.6±1.2	0.961	1.9±0.7	2.1±0.9	0.763
**Triacylglycerole**	TG(16:1_28:0)	0.7±0.4	1.2±1.1	0.452	0.5±0.4	1.5±2.6	0.502
**Triacylglycerole**	TG(16:1_30:1)	1.9±1.7	1.9±1.4	0.988	0.2±0.4	6±9.3	0.323
**Triacylglycerole**	TG(16:1_32:0)	12.9±12.6	16.7±15.7	0.755	2.7±2	52.2±78.5	0.317
**Triacylglycerole**	TG(16:1_32:1)	12.6±12.9	15±16.1	0.848	1.2±1.8	52.3±82.5	0.325
**Triacylglycerole**	TG(16:1_32:2)	4.3±4.1	4.9±5.1	0.89	0.8±0.9	16.1±24.7	0.323
**Triacylglycerole**	TG(16:1_33:1)	0.7±0.7	0.7±0.8	0.909	0.3±0.1	2.2±3	0.299
**Triacylglycerole**	TG(16:1_34:0)	8.8±8.5	9.1±10.1	0.97	1.4±0.6	25.2±37.8	0.318
**Triacylglycerole**	TG(16:1_34:1)	58±56.4	56.8±65	0.982	8.9±3.8	150.7±227.9	0.323
**Triacylglycerole**	TG(16:1_34:2)	45.1±40.5	42.3±51.6	0.945	8±3.7	123.5±182.4	0.315
**Triacylglycerole**	TG(16:1_34:3)	7.3±6.6	7±8.4	0.956	1.7±0.7	24.5±36.1	0.316

**Triacylglycerole**	TG(16:1_36:1)	18±18	14.4±16.8	0.807	1.6±0.5	32.3±49.1	0.321
**Triacylglycerole**	TG(16:1_36:2)	69.1±66.2	50.3±58	0.725	6.6±2.1	126.3±190.1	0.317
**Triacylglycerole**	TG(16:1_36:3)	48.6±44.4	34.5±42.9	0.706	8.2±2.7	99.9±141.4	0.305
**Triacylglycerole**	TG(16:1_36:4)	9.6±7.7	6.7±8.2	0.675	3.5±1.1	21.6±27	0.29
**Triacylglycerole**	TG(16:1_36:5)	0.7±0.6	0.5±0.6	0.729	0.3±0.1	1.5±1.7	0.257
**Triacylglycerole**	TG(16:1_38:3)	2±1.8	1.6±1.8	0.768	0.6±0.2	3.7±4.6	0.285
**Triacylglycerole**	TG(16:1_38:4)	1.4±0.8	1.3±0.9	0.947	1±0.2	2.5±2.3	0.279
**Triacylglycerole**	TG(16:1_38:5)	0.8±0.5	0.8±0.6	0.933	0.7±0.2	1.8±1.5	0.275
**Triacylglycerole**	TG(17:0_32:1)	0.4±0.4	0.4±0.4	0.98	0.3±0.3	1.6±2.1	0.327
**Triacylglycerole**	TG(17:0_34:1)	1.9±1.5	1.8±1.5	0.93	0.9±0.2	5.7±7.1	0.282
**Triacylglycerole**	TG(17:0_34:2)	2±1.7	1.6±1.7	0.783	0.8±0.3	5.1±6	0.265
**Triacylglycerole**	TG(17:0_34:3)	0.5±0.4	0.4±0.4	0.858	0.2±0.2	1±1.2	0.249
**Triacylglycerole**	TG(17:0_36:3)	2.1±1.8	1.4±1.5	0.635	0.9±0.3	4.1±4.1	0.23
**Triacylglycerole**	TG(17:0_36:4)	0.7±0.5	0.6±0.4	0.733	0.6±0.2	1.5±1.1	0.172
**Triacylglycerole**	TG(17:1_32:1)	0.6±0.5	1±0.7	0.471	0.5±0.3	1.8±1.9	0.264
**Triacylglycerole**	TG(17:1_34:1)	2.6±2	2.3±2.2	0.846	0.8±0.4	5.2±6.2	0.261
**Triacylglycerole**	TG(17:1_34:2)	2±1.7	1.7±1.7	0.828	0.9±0.2	4.2±4.8	0.277
**Triacylglycerole**	TG(17:1_34:3)	0.5±0.4	0.4±0.4	0.684	0.3±0.1	1.1±1.1	0.207
**Triacylglycerole**	TG(17:1_36:3)	2.5±2.3	1.5±1.7	0.571	0.7±0.2	4±4.6	0.258
**Triacylglycerole**	TG(17:1_36:4)	0.7±0.4	0.5±0.5	0.557	0.5±0.1	1.5±1.3	0.229
**Triacylglycerole**	TG(17:1_38:5)	0.4±0.1	0.3±0.1	0.136	0.4±0.1	0.6±0.1	0.055
**Triacylglycerole**	TG(17:1_38:6)	0.3±0.1	0.2±0.1	0.43	0.2±0.1	0.3±0.1	0.148
**Triacylglycerole**	TG(17:1_38:7)	0.1±0.1	0.1±0.1	0.177	0.1±0.1	0.1±0.1	0.258
**Triacylglycerole**	TG(17:2_34:2)	0.3±0.2	0.2±0.2	0.597	0.2±0.1	0.6±0.6	0.235
**Triacylglycerole**	TG(17:2_36:2)	0.5±0.4	0.4±0.3	0.556	0.3±0.1	0.9±1	0.306
**Triacylglycerole**	TG(17:2_36:3)	0.5±0.4	0.4±0.3	0.545	0.3±0.1	1±1.1	0.244
**Triacylglycerole**	TG(17:2_36:4)	0.5±0.3	0.4±0.3	0.632	0.4±0.1	0.8±0.5	0.214
**Triacylglycerole**	TG(17:2_38:5)	0.3±0.2	0.3±0.2	0.608	0.3±0.1	0.5±0.2	0.069
**Triacylglycerole**	TG(17:2_38:6)	0.4±0.2	0.3±0.2	0.512	0.2±0.1	0.5±0.3	0.183
**Triacylglycerole**	TG(17:2_38:7)	0.1±0.1	0.1±0.1	0.593	0.1±0.1	0.2±0.2	0.331
**Triacylglycerole**	TG(18:0_30:0)	1.5±1.3	1.6±1.6	0.912	0.5±0.2	2.1±2.9	0.359
**Triacylglycerole**	TG(18:0_30:1)	1.3±1.1	1.6±1.5	0.778	0.5±0.3	2.6±3.7	0.352
**Triacylglycerole**	TG(18:0_32:0)	4.8±4.3	5±5	0.968	1.9±0.7	7.6±7.5	0.239
**Triacylglycerole**	TG(18:0_32:1)	10.1±9.9	9.8±11.1	0.977	1.5±0.4	22.2±33.3	0.322
**Triacylglycerole**	TG(18:0_32:2)	5.3±5.1	4.7±5.7	0.906	0.9±0.2	10.3±14.6	0.307
**Triacylglycerole**	TG(18:0_34:2)	40.2±37.2	31.4±38.5	0.787	8.9±2	83.3±116.2	0.31
**Triacylglycerole**	TG(18:0_34:3)	8.2±7.3	6.5±8.3	0.799	1.6±0.5	17.5±24.9	0.311
**Triacylglycerole**	TG(18:0_36:1)	11.8±11.5	9.4±11.8	0.805	1.6±0.3	17.1±24	0.304
**Triacylglycerole**	TG(18:0_36:2)	55.9±53.3	42.3±53.1	0.764	7.3±0.9	79.3±113.9	0.316

**Triacylglycerole**	TG(18:0_36:3)	52.9±49.7	36±46.5	0.682	11.9±1.1	84.7±113.2	0.308
**Triacylglycerole**	TG(18:0_36:4)	14.6±12.3	9.6±11.5	0.627	6±0.9	28.1±33.3	0.293
**Triacylglycerole**	TG(18:0_36:5)	1.1±0.8	1±1	0.884	0.6±0.1	2.1±2.3	0.295
**Triacylglycerole**	TG(18:0_38:6)	1.3±0.5	1.4±0.9	0.897	1.7±0.3	2.1±0.3	0.077
**Triacylglycerole**	TG(18:0_38:7)	0.3±0.1	0.3±0.2	0.574	0.2±0.1	0.5±0.3	0.112
**Triacylglycerole**	TG(18:1_26:0)	2.5±1.3	2.7±1.2	0.846	1.2±0.3	2.8±2.9	0.365
**Triacylglycerole**	TG(18:1_28:1)	2.5±0.8	1.9±1	0.403	0.5±0.4	3.2±3.9	0.283
**Triacylglycerole**	TG(18:1_30:0)	18.3±16.8	21.5±20.2	0.837	3.6±1.3	34.2±46.2	0.296
**Triacylglycerole**	TG(18:1_30:1)	8.8±7.9	10±7.7	0.86	2±1.5	18±27.2	0.347
**Triacylglycerole**	TG(18:1_30:2)	2.9±1.4	2.8±1.4	0.921	1.2±0.6	4.6±6.1	0.372
**Triacylglycerole**	TG(18:1_32:0)	90.7±83.1	99.1±99.9	0.914	29.5±9.3	212.2±271.7	0.289
**Triacylglycerole**	TG(18:1_32:1)	81.6±77.9	75.5±85.8	0.931	10.7±3.3	173±258.3	0.319
**Triacylglycerole**	TG(18:1_32:2)	28.4±25.5	23.8±28.8	0.843	4.2±1.2	57.2±84.3	0.318
**Triacylglycerole**	TG(18:1_32:3)	2±1.6	1.5±1.6	0.724	0.5±0.2	4±5.2	0.285
**Triacylglycerole**	TG(18:1_33:0)	1.9±1.5	1.9±1.6	0.972	0.9±0.1	3.7±4.1	0.286
**Triacylglycerole**	TG(18:1_33:1)	4.3±3.8	3.7±3.5	0.854	1.8±0.6	8.7±9.7	0.263
**Triacylglycerole**	TG(18:1_33:2)	2.3±1.8	1.6±1.8	0.601	1±0.3	4.7±4.7	0.216
**Triacylglycerole**	TG(18:1_33:3)	0.5±0.3	0.3±0.3	0.38	0.2±0.1	0.7±0.6	0.13
**Triacylglycerole**	TG(18:1_34:1)	371.8±351.9	324.8±386.4	0.882	61.8±10	604.7±828.3	0.3
**Triacylglycerole**	TG(18:1_34:2)	293.5±268.4	239.1±301.1	0.824	61.1±10.6	548±740.2	0.299
**Triacylglycerole**	TG(18:1_34:3)	50.9±47.1	38.9±49.6	0.771	10.2±2.9	108.8±152.5	0.306
**Triacylglycerole**	TG(18:1_34:4)	2.2±1.8	1.6±2	0.713	0.8±0.2	4.9±6	0.275
**Triacylglycerole**	TG(18:1_35:2)	6±5.5	4.1±4.3	0.652	1.5±0.3	10.6±12.3	0.247
**Triacylglycerole**	TG(18:1_35:3)	2.5±2.1	1.6±1.9	0.593	0.8±0.3	4.3±5.1	0.273
**Triacylglycerole**	TG(18:1_36:0)	12.8±12	9.8±11.3	0.758	2±0.5	17.5±24.2	0.309
**Triacylglycerole**	TG(18:1_36:1)	129.6±124.8	92±112.9	0.712	12.9±2	164.6±235.3	0.307
**Triacylglycerole**	TG(18:1_36:2)	423.1±406.7	274.6±330.5	0.641	47.5±12.6	501.9±691.2	0.299
**Triacylglycerole**	TG(18:1_36:3)	361.1±336.1	204.8±262.4	0.549	66.5±9.3	546.6±721.9	0.294
**Triacylglycerole**	TG(18:1_36:4)	72.7±63.9	42±53.1	0.546	27.1±3.9	136.6±157.4	0.274
**Triacylglycerole**	TG(18:1_36:5)	4.3±3.5	2.7±3.3	0.59	2±0.2	7.9±8.3	0.261
**Triacylglycerole**	TG(18:1_36:6)	0.5±0.2	0.4±0.3	0.472	0.4±0.1	0.9±0.6	0.213
**Triacylglycerole**	TG(18:1_38:5)	5±3.4	4.2±3.2	0.768	3.9±0.5	8.6±5.1	0.165
**Triacylglycerole**	TG(18:1_38:6)	3.5±1.8	3.4±2.1	0.969	3.3±0.8	4.9±2.2	0.245
**Triacylglycerole**	TG(18:1_38:7)	0.8±0.5	0.7±0.5	0.835	0.5±0.2	1.7±1.9	0.276
**Triacylglycerole**	TG(18:2_28:0)	3±1.5	3.3±1.6	0.825	0.5±0.2	4.7±4.8	0.183
**Triacylglycerole**	TG(18:2_30:0)	13.3±10.7	15.6±16	0.841	4.9±1.3	26.6±30.4	0.263
**Triacylglycerole**	TG(18:2_30:1)	4.6±3.2	4.5±3.8	0.967	1.1±0.4	8.9±11.9	0.301
**Triacylglycerole**	TG(18:2_32:0)	67±53.1	76.4±80.8	0.872	33.9±10.9	176.5±198.2	0.26
**Triacylglycerole**	TG(18:2_32:1)	38.9±34.4	36.1±44.4	0.933	8.8±3.4	84.4±115	0.299

**Triacylglycerole**	TG(18:2_32:2)	10.4±9.5	9±11.4	0.867	3.1±1.2	22.5±29.5	0.299
**Triacylglycerole**	TG(18:2_33:0)	1.5±1.1	1.4±1.3	0.979	1.1±0.2	3.6±3.1	0.21
**Triacylglycerole**	TG(18:2_33:1)	2.6±2.2	2.1±2.1	0.804	1.6±0.5	5.5±5.1	0.226
**Triacylglycerole**	TG(18:2_33:2)	1.1±0.7	0.9±0.9	0.788	0.8±0.1	2.6±2.1	0.188
**Triacylglycerole**	TG(18:2_34:0)	37.8±33.6	31.5±39.8	0.844	11.4±2.3	77.6±101.6	0.302
**Triacylglycerole**	TG(18:2_34:1)	184.1±166	150.8±195.4	0.83	53.2±10.5	336.4±419.1	0.287
**Triacylglycerole**	TG(18:2_34:2)	109.7±94.6	82.8±109.3	0.758	42.7±11.2	233.5±279.4	0.282
**Triacylglycerole**	TG(18:2_34:3)	15.1±13	11.5±15.1	0.762	6±1.6	36.3±44	0.279
**Triacylglycerole**	TG(18:2_34:4)	0.8±0.7	0.7±0.7	0.771	0.7±0.2	2.1±1.8	0.231
**Triacylglycerole**	TG(18:2_35:1)	3±2.7	2±2.3	0.637	1.3±0.3	5.6±6	0.253
**Triacylglycerole**	TG(18:2_35:2)	2.7±2.3	1.8±2	0.63	1.3±0.2	5.3±5.5	0.246
**Triacylglycerole**	TG(18:2_35:3)	1±0.8	0.6±0.6	0.446	0.6±0.2	2±1.6	0.179
**Triacylglycerole**	TG(18:2_36:0)	8.5±7.6	6.2±8	0.725	2.2±0.3	14.6±19	0.301
**Triacylglycerole**	TG(18:2_36:1)	62.2±57.9	40.7±53.7	0.655	13.9±1.7	103.9±139.2	0.306
**Triacylglycerole**	TG(18:2_36:2)	175.2±159.9	107.2±140.2	0.6	40.2±6.3	282.4±365.5	0.295
**Triacylglycerole**	TG(18:2_36:3)	109.9±98.7	58.8±78	0.508	41.9±4.8	211.5±244.6	0.275
**Triacylglycerole**	TG(18:2_36:4)	24.1±19.7	13.8±16.5	0.51	16.9±3.4	51.3±48	0.262
**Triacylglycerole**	TG(18:2_36:5)	1.7±1.2	1.1±1.3	0.573	1.5±0.3	3.4±2.6	0.246
**Triacylglycerole**	TG(18:2_38:4)	4.2±2.3	3.4±2.6	0.695	4.3±0.7	7.8±3.5	0.142
**Triacylglycerole**	TG(18:2_38:5)	3±1.6	2.3±1.6	0.599	3.3±0.7	5.2±2.1	0.16
**Triacylglycerole**	TG(18:2_38:6)	2.2±0.9	1.8±1.1	0.68	2.7±1	2.8±1	0.889
**Triacylglycerole**	TG(18:3_30:0)	0.6±0.4	0.9±0.6	0.45	0.4±0.1	1.3±1.1	0.175
**Triacylglycerole**	TG(18:3_32:0)	2.4±1.7	3.6±2.9	0.567	1.8±0.7	7±6.2	0.196
**Triacylglycerole**	TG(18:3_32:1)	1.2±1.1	1.5±1.7	0.79	0.6±0.2	3.5±4.1	0.257
**Triacylglycerole**	TG(18:3_34:0)	1.7±1.3	1.5±1.8	0.901	0.7±0.2	3.5±3.8	0.259
**Triacylglycerole**	TG(18:3_34:1)	7.9±6.3	7.4±8.3	0.933	3.4±0.9	16.5±18.2	0.259
**Triacylglycerole**	TG(18:3_34:2)	4.2±3.1	3.8±4.6	0.9	2.1±0.4	10.3±11.1	0.248
**Triacylglycerole**	TG(18:3_34:3)	0.8±0.5	0.6±0.6	0.6	0.4±0.2	1.5±1.5	0.241
**Triacylglycerole**	TG(18:3_35:2)	0.2±0.2	0.2±0.2	0.645	0.1±0.1	0.4±0.4	0.196
**Triacylglycerole**	TG(18:3_36:1)	2.9±2.3	2±2.5	0.673	1±0.3	5±6.2	0.309
**Triacylglycerole**	TG(18:3_36:2)	7.5±6.5	4.7±5.9	0.602	2.5±0.4	12.4±14.5	0.279
**Triacylglycerole**	TG(18:3_36:3)	4.1±3.3	2.7±3.2	0.623	2.1±0.3	8±8	0.249
**Triacylglycerole**	TG(18:3_36:4)	1.3±0.7	0.8±0.9	0.519	1±0.3	2.1±1.6	0.266
**Triacylglycerole**	TG(18:3_38:5)	0.2±0.1	0.2±0.2	0.999	0.3±0.1	0.4±0.2	0.223
**Triacylglycerole**	TG(18:3_38:6)	0.3±0.1	0.3±0.2	0.735	0.3±0.1	0.4±0.2	0.456
**Triacylglycerole**	TG(20:0_32:3)	0.6±0.5	0.5±0.6	0.806	0.3±0.1	1.1±1.2	0.259
**Triacylglycerole**	TG(20:0_32:4)	0.5±0.4	0.4±0.5	0.858	0.3±0.1	0.9±0.9	0.268
**Triacylglycerole**	TG(20:1_30:1)	0.5±0.2	0.3±0.2	0.141	0.2±0.1	0.5±0.6	0.421
**Triacylglycerole**	TG(20:1_32:1)	1.7±1.6	1.6±1.7	0.953	0.4±0.1	2.8±3.9	0.322

**Triacylglycerole**	TG(20:1_32:2)	1.2±1.2	0.9±1	0.765	0.3±0.2	1.7±2.2	0.31
**Triacylglycerole**	TG(20:1_32:3)	0.3±0.2	0.3±0.3	0.988	0.1±0.1	0.6±0.7	0.277
**Triacylglycerole**	TG(20:1_34:0)	1.3±1	1.2±1.2	0.928	0.6±0.1	2±2.2	0.32
**Triacylglycerole**	TG(20:1_34:1)	7.5±6.9	6.6±7.5	0.876	2.2±0.3	10.9±13.9	0.32
**Triacylglycerole**	TG(20:1_34:2)	7.4±6.5	5.6±6.8	0.747	2±0.5	12.4±15.6	0.292
**Triacylglycerole**	TG(20:1_34:3)	1.6±1.4	1.2±1.4	0.748	0.4±0.1	3±4.2	0.309
**Triacylglycerole**	TG(20:2_32:0)	0.7±0.7	0.9±0.5	0.703	1±0.4	2.2±1.6	0.23
**Triacylglycerole**	TG(20:2_32:1)	0.8±0.6	0.8±0.8	0.955	0.5±0.2	1.5±1.7	0.335
**Triacylglycerole**	TG(20:2_34:1)	2.7±2.1	2.4±2	0.857	1.7±0.4	5.3±4.9	0.244
**Triacylglycerole**	TG(20:2_34:2)	2.3±2	1.8±2	0.743	1.5±0.5	4.9±5.3	0.302
**Triacylglycerole**	TG(20:2_34:3)	0.6±0.4	0.5±0.5	0.659	0.3±0.1	1.3±1.4	0.263
**Triacylglycerole**	TG(20:2_34:4)	0.1±0.1	0.1±0.1	0.649	0.1±0.1	0.1±0.1	0.442
**Triacylglycerole**	TG(20:2_36:5)	0.2±0.1	0.2±0.1	0.954	0.2±0.1	0.2±0.1	0.19
**Triacylglycerole**	TG(20:3_32:0)	1.4±0.6	2.3±1.4	0.325	1.7±0.5	3.4±1	0.036*§
**Triacylglycerole**	TG(20:3_32:1)	1±0.6	1±0.8	0.968	0.5±0.2	1.8±1.5	0.174
**Triacylglycerole**	TG(20:3_32:2)	0.5±0.2	0.3±0.2	0.476	0.3±0.2	0.7±0.5	0.215
**Triacylglycerole**	TG(20:3_34:0)	0.7±0.5	0.9±0.6	0.71	0.7±0.2	1.4±0.8	0.194
**Triacylglycerole**	TG(20:3_34:1)	3.5±2.4	3.7±3	0.941	2.4±0.3	6.5±4.5	0.164
**Triacylglycerole**	TG(20:3_34:2)	2.9±2.1	2.6±2.6	0.894	2±0.5	5.9±4.5	0.187
**Triacylglycerole**	TG(20:3_34:3)	0.7±0.5	0.5±0.4	0.491	0.5±0.2	1.1±0.9	0.203
**Triacylglycerole**	TG(20:3_36:3)	2.8±2.2	2.1±2.1	0.71	1.8±0.1	4.9±3.6	0.186
**Triacylglycerole**	TG(20:3_36:4)	0.9±0.6	0.8±0.6	0.835	0.9±0.2	1.6±0.8	0.188
**Triacylglycerole**	TG(20:3_36:5)	0.1±0.1	0.2±0.1	0.583	0.1±0.1	0.2±0.1	0.036*§
**Triacylglycerole**	TG(20:4_30:0)	1.4±0.3	2.8±1.4	0.13	1.5±0.3	2.1±0.3	0.021*
**Triacylglycerole**	TG(20:4_32:0)	7.4±1.7	14.6±8.1	0.181	8.8±1.8	13.2±0.8	0.007*§
**Triacylglycerole**	TG(20:4_32:1)	2.1±1.2	3.2±2.1	0.452	1.9±0.8	4.1±3.1	0.261
**Triacylglycerole**	TG(20:4_32:2)	0.9±0.4	0.9±0.7	0.943	0.8±0.2	1.4±0.8	0.294
**Triacylglycerole**	TG(20:4_33:2)	0.2±0.1	0.2±0.1	0.934	0.2±0.1	0.3±0.1	0.334
**Triacylglycerole**	TG(20:4_34:0)	3.5±1.6	5.1±3	0.445	3.8±0.7	5.9±1.5	0.067
**Triacylglycerole**	TG(20:4_34:1)	10.5±5.9	12.7±9.3	0.735	10.1±2.3	17.6±9.4	0.227
**Triacylglycerole**	TG(20:4_34:2)	8.7±4.6	8.5±6.7	0.972	9.2±2.4	14.4±7.4	0.29
**Triacylglycerole**	TG(20:4_34:3)	1±0.6	1.2±1	0.853	1.2±0.4	1.8±1.2	0.366
**Triacylglycerole**	TG(20:4_35:3)	0.1±0.1	0.1±0.1	0.622	0.1±0.1	0.2±0.1	0.327
**Triacylglycerole**	TG(20:4_36:2)	8±5.1	7.1±5.5	0.831	7.3±1.4	12.4±7.1	0.269
**Triacylglycerole**	TG(20:4_36:3)	6.1±3.9	4.9±3.9	0.715	6.3±1.2	9.1±4.2	0.302
**Triacylglycerole**	TG(20:4_36:4)	4.1±1.3	3.8±2.5	0.854	4.9±1	4.6±1.6	0.778
**Triacylglycerole**	TG(20:4_36:5)	0.4±0.2	0.4±0.3	0.962	0.4±0.2	0.4±0.2	0.917
**Triacylglycerole**	TG(20:5_34:0)	0.2±0.1	0.2±0.1	0.82	0.2±0.1	0.2±0.1	0.428
**Triacylglycerole**	TG(20:5_34:1)	0.7±0.2	0.8±0.3	0.786	0.9±0.1	1.3±0.6	0.276

**Triacylglycerole**	TG(20:5_34:2)	0.4±0.2	0.4±0.2	0.912	0.4±0.1	0.9±0.7	0.275
**Triacylglycerole**	TG(20:5_36:2)	0.6±0.2	0.5±0.3	0.562	0.6±0.1	1±0.9	0.42
**Triacylglycerole**	TG(20:5_36:3)	0.4±0.2	0.4±0.3	0.758	0.4±0.1	0.9±0.8	0.243
**Triacylglycerole**	TG(22:0_32:4)	0.1±0.1	0.1±0.1	0.612	0.1±0.1	0.2±0.2	0.195
**Triacylglycerole**	TG(22:2_32:4)	0.2±0.2	0.2±0.2	0.649	0.2±0.1	0.5±0.3	0.129
**Triacylglycerole**	TG(22:4_32:0)	0.7±0.3	1.3±0.8	0.251	0.9±0.2	1.6±0.4	0.014*§
**Triacylglycerole**	TG(22:4_32:2)	0.1±0.1	0.1±0.1	0.916	0.1±0.1	0.2±0.2	0.308
**Triacylglycerole**	TG(22:4_34:2)	1±0.7	1.1±1.1	0.902	1.1±0.3	2.2±1.1	0.126
**Triacylglycerole**	TG(22:5_32:0)	2.6±0.7	4.1±1.1	0.066	2.5±0.4	4.5±1.3	0.039*§
**Triacylglycerole**	TG(22:5_32:1)	0.8±0.4	1.1±0.5	0.441	0.7±0.1	1.7±0.9	0.096
**Triacylglycerole**	TG(22:5_34:1)	2.9±1.7	3.1±1.6	0.864	2.6±0.3	4.9±2.3	0.125
**Triacylglycerole**	TG(22:5_34:2)	2.4±1	2.4±1.3	0.982	2.6±0.6	3.9±1.8	0.272
**Triacylglycerole**	TG(22:5_34:3)	0.3±0.2	0.5±0.3	0.372	0.5±0.2	0.8±0.4	0.243
**Triacylglycerole**	TG(22:6_32:0)	10.2±2.1	15.8±7.1	0.235	6.5±1.6	10.7±2	0.028*§
**Triacylglycerole**	TG(22:6_32:1)	2.3±0.9	2.9±1.7	0.578	1.8±0.7	3.1±1.6	0.237
**Triacylglycerole**	TG(22:6_34:1)	8.4±3.7	10±5.6	0.691	7.5±2.3	10.2±2.9	0.241
**Triacylglycerole**	TG(22:6_34:2)	6.2±2.6	5.8±3.5	0.879	6.4±2.6	6.9±2.5	0.824
**Triacylglycerole**	TG(22:6_34:3)	0.7±0.2	0.8±0.5	0.867	0.8±0.3	0.9±0.4	0.539

9.2 High fat diet

class	Metabolite	WT HFD	*Cndp1*-KO HFD	*p* (vs WT HFD)	WT HFD + STZ	Cndp1-KO HFD + STZ	*p* (vs WT HFD + STZ)
**Amino acid**	Alanine	390.2±181.5	542.1±79.9	0.118	568.4±87.0	591.9±105.7	0.71
**Amino acid**	Arginine	68.8±9.2	76.4±17.4	0.407	125.9±33.3	129.4±16.7	0.841
**Amino acid**	Asparagine	99.8±27.1	86.6±23.3	0.428	87.8±19.0	104.4±19.7	0.206
**Amino acid**	Aspartic acid	598.9±113.1	681.4±232.4	0.492	435.2±148.7	524.6±159.3	0.381
**Amino acid**	Glutamine	340.6±87.0	377.3±66.2	0.47	376.3±43.3	427.7±102.5	0.326
**Amino acid**	Glutamate	1426.8±192.5	1968.7±269.3	0.005*	1878.8±281.1	1984.1±246.0	0.543
**Amino acid**	Glycine	1729.5±271.5	2150.5±354.5	0.062	2040.3±333.7	1938.2±434.1	0.686
**Amino acid**	Histidine	81.4±11.1	97.1±24.0	0.215	106.7±21.6	109.0±28.1	0.888
**Amino acid**	Isoleucine	56.9±9.3	74.0±21.6	0.135	87.6±19.8	87.5±14.1	0.995
**Amino acid**	Leucine	49.3±49.4	135.7±29.4	0.008*	182.2±43.8	173.6±28.0	0.72
**Amino acid**	Lysine	99.2±13.7	120.5±21.0	0.088	100.6±11.0	101.5±15.7	0.918
**Amino acid**	Methionine	47.5±6.7	62.6±12.0	0.034*	67.9±10.3	80.7±27.7	0.356
**Amino acid**	Phenylalanine	54.8±8.5	66.9±18.6	0.215	69.2±12.1	71.3±11.9	0.793
**Amino acid**	Proline	69.8±11.3	80.7±18.4	0.282	100.4±23.6	109.2±17.3	0.514
**Amino acid**	Serine	402.9±190.0	588.9±121.9	0.096	519.6±77.9	575.4±99.1	0.346
**Amino acid**	Tyrosine	65.1±10.5	80.5±24.5	0.227	85.1±15.1	89.9±18.0	0.659
**Amino acid**	Valine	114.3±22.2	130.5±64.6	0.606	165.6±33.3	161.9±23.5	0.844
**Acylcarnitines**	L-Carnitine	234.8±22.4	255.5±40.8	0.469	221.8±27.9	288.5±37.3	0.048*
**Acylcarnitines**	L-Acetylcarnitine	55.2±7.9	63.8±8.2	0.24	69.9±12	77.5±24.8	0.647
**Acylcarnitines**	Propionylcarnitine	2.3±0.5	2.4±0.3	0.627	2.8±1.3	3.4±1.4	0.606
**Acylcarnitines**	Malonylcarnitine	0.6±0.1	0.7±0.2	0.199	1.6±0.7	1.6±0.8	0.886
**Acylcarnitines**	Butyrylcarnitine	2.9±0.4	3.8±0.2	0.01*	4.6±1.3	5.9±1.7	0.326
**Acylcarnitines**	Butenylcarnitine	0.1±0.1	0.1±0.1	0.809	0.1±0.1	0.2±0.1	0.304
**Acylcarnitines**	2-Methylbutyroylcarnitine	0.3±0.1	0.3±0.2	0.829	0.5±0.3	0.5±0.4	0.746
**Acylcarnitines**	Glutarylcarnitine	0.4±0.1	0.4±0.1	0.91	0.6±0.1	0.5±0.1	0.191
**Acylcarnitines**	3-Methylglutarylcarnitine	0.2±0.1	0.2±0.1	0.507	0.2±0.1	0.2±0.1	0.653
**Acylcarnitines**	Methylmalonylcarnitine	0.9±0.2	0.9±0.2	0.776	1.1±0.3	1.2±0.2	0.579
**Acylcarnitines**	Glutaconylcarnitine	0.1±0.1	0.1±0.1	0.791	0.1±0.1	0.1±0.1	0.201
**Acylcarnitines**	Hexanoylcarnitine	0.9±0.2	1.1±0.2	0.147	1±0.3	1.2±0.2	0.359
**Acylcarnitines**	L-Octanoylcarnitine	0.2±0.2	0.3±0.2	0.503	0.3±0.2	0.6±0.1	0.021*§
**Acylcarnitines**	Nonanoylcarnitine	0.1±0.1	0.1±0.1	0.643	0.1±0.1	0.2±0.1	0.303
**Acylcarnitines**	Dodecanoylcarnitine	1.2±0.2	2±0.5	0.026*§	2.1±1	2.2±0.5	0.912
**Acylcarnitines**	Tetradecanoylcarnitine	2.7±0.4	3.7±0.6	0.046*	4±1.6	4.7±0.9	0.502
**Acylcarnitines**	cis-5-Tetradecenoylcarnitine	0.3±0.1	0.4±0.1	0.341	0.3±0.1	0.4±0.1	0.268
**Acylcarnitines**	3-Hydroxy-cis-5-tetradecenoylcarnitine	0.1±0.1	0.2±0.1	0.144	0.2±0.1	0.2±0.1	0.077
**Acylcarnitines**	O-(hydroxytetradecadienoyl)-L-carnitine	0.1±0.1	0.1±0.1	0.315	0.1±0.1	0.1±0.1	0.031*§

**Acylcarnitines**	L-Palmitoylcarnitine	7.4±0.9	8.8±0.8	0.079	8±2.3	9.6±1.6	0.361
**Acylcarnitines**	3-Hydroxyhexadecanoylcarnitine	0.3±0.1	0.3±0.1	0.532	0.3±0.1	0.4±0.1	0.02*§
**Acylcarnitines**	9-Hexadecenoylcarnitine	0.7±0.1	0.9±0.2	0.042*	0.7±0.2	0.7±0.2	0.895
**Acylcarnitines**	3-Hydroxy-9-hexadecenoylcarnitine	0.1±0.1	0.1±0.1	0.621	0.1±0.1	0.1±0.1	0.81
**Acylcarnitines**	9,12-Hexadecadienoylcarnitine	0.3±0.1	0.3±0.1	0.545	0.3±0.1	0.4±0.1	0.096
**Acylcarnitines**	Stearoylcarnitine	3.9±0.4	4.4±0.6	0.177	3.9±0.8	4.5±0.8	0.35
**Acylcarnitines**	Oleoylcarnitine	2.5±0.3	3.1±0.3	0.033*	3±0.8	2.8±0.5	0.654
**Acylcarnitines**	3-Hydroxy-11Z-octadecenoylcarnitine	0.3±0.1	0.3±0.1	0.475	0.3±0.1	0.4±0.1	0.044*
**Acylcarnitines**	Linoleyl carnitine	1±0.2	1.3±0.2	0.015*	1.4±0.5	1.6±0.4	0.46
**Amino acid related**	1-Methylhistidine	23.4±9.4	18.8±2.4	0.44	34.7±6.2	28.5±5.1	0.223
**Amino acid related**	3-Methylhistidine	4.2±1.1	1.4±0.1	0.004*§	4.1±0.9	1.4±0.3	0.003*§
**Amino acid related**	5-Aminopentanoic acid	0.8±0.4	1±0.2	0.299	1.1±0.4	1.6±0.7	0.298
**Amino acid related**	L-alpha-Aminobutyric acid	34.7±9.2	29.6±11.1	0.562	40.6±18.7	40.9±11.7	0.987
**Amino acid related**	Asymmetric dimethylarginine	4.7±0.5	4.8±2.3	0.966	6.8±3.3	5.2±3.4	0.566
**Amino acid related**	Aminoadipic acid	24.5±13.2	24.5±10.5	0.999	25.5±8	45±27.5	0.282
**Amino acid related**	3-Aminobutanoic acid	4.9±1.3	4.4±2.9	0.794	26.4±20.3	28.9±22.7	0.89
**Amino acid related**	Betaine	1384.5±473.5	1336.3±357.3	0.893	2272±784.7	2872.8±1569.5	0.575
**Amino acid related**	Citrulline	25.4±6.6	24.4±3.6	0.824	34±4.1	34.6±7.9	0.914
**Amino acid related**	Creatinine	34.4±11.7	38.4±2.8	0.587	71.8±86.8	28.3±9.6	0.421
**Amino acid related**	Cystine	427.5±135.6	443.9±496.8	0.958	777±282	895.3±670.3	0.788
**Amino acid related**	Homo-L-arginine	0.3±0.1	0.3±0.1	0.899	0.3±0.1	0.4±0.3	0.374
**Amino acid related**	Homocysteine	46.3±12.6	65.9±18	0.171	54.9±18.2	46.4±9.8	0.506
**Amino acid related**	Ornithine	14.5±4.9	18.1±5.3	0.408	24.8±3	31±8.3	0.267
**Amino acid related**	Phenylacetylglycine	16.8±4.8	25.3±11.5	0.282	49.9±60.6	15.3±6.3	0.363
**Amino acid related**	L-phenylalanine betaine	0.1±0.1	0.1±0.1	0.191	0.1±0.1	0.1±0.1	0.671
**Amino acid related**	Proline betaine	1.7±0.3	2.2±0.3	0.088	1.8±0.3	1.5±0.2	0.05*
**Amino acid related**	Sarcosine	1.3±0.3	1.4±0.5	0.802	4±1.3	4.4±1.3	0.748
**Amino acid related**	Symmetric dimethylarginine	0.9±0.2	0.9±0.3	0.988	1.2±1.2	0.7±0.2	0.516
**Amino acid related**	4-Hydroxyproline	28.2±4.9	26.9±4.8	0.753	30.2±7.7	33.2±9.5	0.68
**Amino acid related**	Taurine	3041.8±199.8	3232.5±334.6	0.43	3317.8±141.5	3116.8±162.8	0.158
**Bile acids**	Cholic acid	0.1±0.1	0.1±0.1	0.792	1.1±0.8	2.1±3	0.591
**Bile acids**	Deoxycholic acid	0.2±0.1	0.2±0.2	0.974	0.4±0.3	0.7±0.4	0.344
**Bile acids**	Taurocholic acid	2.9±2.9	2.6±3.6	0.909	8.5±2.6	7.5±3.6	0.707
**Bile acids**	Taurochenodesoxycholic acid	0.1±0.1	0.1±0.1	0.846	0.3±0.1	0.2±0.1	0.496
**Bile acids**	Taurodeoxycholic acid	0.3±0.2	0.2±0.2	0.52	0.3±0.2	0.5±0.2	0.195
**Bile acids**	Tauro-b-muricholic acid	0.8±0.8	1.8±2.4	0.492	4.9±3.8	1.5±1.2	0.193
**biogenic amines**	beta-Alanine	30.3±2.7	25.3±4	0.118	88.5±44.6	49.1±9.5	0.185
**biogenic amines**	gamma-Aminobutyric acid	60.6±5.2	72.8±12.4	0.167	42.1±12.3	46.3±17.9	0.746
**biogenic amines**	Histamine	3.5±1.5	4.5±1.1	0.389	0.7±0.4	2.4±3	0.383

**biogenic amines**	Putrescine	8.6±2.1	7.9±1.9	0.693	8.7±3.2	8.5±2.4	0.918
**biogenic amines**	Serotonin	2.1±1.1	2.3±0.5	0.766	1.5±0.9	2.3±1.1	0.385
**biogenic amines**	Spermidine	8.3±1.3	9.8±0.8	0.13	11±0.9	13±4	0.423
**biogenic amines**	Spermine	4.6±1.1	5.8±2	0.369	4.3±1.8	6.7±4.9	0.465
**Carboxylic acids**	cis-Aconitic acid	1±0.4	1.1±0.4	0.673	1.9±1.4	1.2±0.4	0.434
**Carboxylic acids**	Dodecanedioic acid	1.6±0.6	1.9±0.8	0.568	1.9±0.3	2.1±0.4	0.363
**Carboxylic acids**	Hippuric acid	0.2±0.1	0.6±0.5	0.213	0.8±0.7	0.8±0.6	0.962
**Carboxylic acids**	L-Lactic acid	9468±2401.4	11119±2073	0.403	15067.8±2048.1	16941.3±2589.4	0.364
**Carboxylic acids**	3-Hydroxyglutaric acid	15.3±3.1	18.5±4.1	0.305	11.4±2.8	18.6±6.6	0.13
**Carboxylic acids**	Succinic acid	115.1±15	140.2±28.1	0.221	170.5±30.8	229±120.8	0.448
**Ceramides**	Cer(d16:1/22:0)	0.3±0.1	0.3±0.1	0.537	0.3±0.1	0.4±0.1	0.75
**Ceramides**	Cer(d16:1/24:0)	0.4±0.1	0.4±0.1	0.947	0.4±0.1	0.4±0.2	0.657
**Ceramides**	Cer(d18:1/14:0)	0.7±0.2	0.7±0.1	0.984	0.8±0.2	0.8±0.2	0.943
**Ceramides**	Cer(d18:1/16:0)	5.7±1	6.2±0.5	0.417	7±1.8	7±1	0.972
**Ceramides**	Cer(d18:1/18:0(OH))	0.4±0.2	0.5±0.1	0.649	0.5±0.2	0.7±0.2	0.288
**Ceramides**	Cer(d18:1/18:0)	1.1±0.1	1.4±0.3	0.071	1.3±0.3	1.2±0.3	0.871
**Ceramides**	Cer(d18:1/18:1)	0.2±0.1	0.2±0.1	0.088	0.2±0.1	0.2±0.1	0.163
**Ceramides**	Cer(d18:1/20:0(OH))	27.7±2.8	30.9±2.6	0.201	40.1±4.2	38.2±8.8	0.749
**Ceramides**	Cer(d18:1/20:0)	1.2±0.1	1.4±0.1	0.071	1.5±0.2	1.4±0.3	0.876
**Ceramides**	Cer(d18:1/22:0)	8.7±0.6	9.5±0.6	0.143	10.2±1.4	10.9±1.4	0.564
**Ceramides**	Cer(d18:1/23:0)	3.1±0.5	3.3±0.4	0.581	3.7±0.5	3.8±0.4	0.638
**Ceramides**	Cer(d18:1/24:0)	19±1.2	20.7±1.7	0.21	18.2±2.9	21.8±2.5	0.146
**Ceramides**	Cer(d18:1/24:1)	9±0.7	9.8±0.5	0.117	10.7±1.6	9.8±1	0.454
**Ceramides**	Cer(d18:1/25:0)	1±0.2	1.1±0.2	0.692	1.3±0.3	1.3±0.3	0.766
**Ceramides**	Cer(d18:1/26:0)	0.2±0.1	0.3±0.1	0.003*[§]	0.2±0.1	0.2±0.1	0.163
**Ceramides**	Cer(d18:1/26:1)	0.2±0.1	0.2±0.1	0.334	0.2±0.1	0.2±0.1	0.44
**Ceramides**	Cer(d18:2/14:0)	0.1±0.1	0.1±0.1	0.78	0.1±0.1	0.1±0.1	0.548
**Ceramides**	Cer(d18:2/16:0)	3.8±0.7	3.8±0.6	0.989	5.3±1.8	4.8±1.2	0.715
**Ceramides**	Cer(d18:2/18:0)	0.4±0.1	0.4±0.1	0.426	0.4±0.2	0.4±0.1	0.973
**Ceramides**	Cer(d18:2/20:0)	0.4±0.1	0.4±0.1	0.961	0.4±0.1	0.4±0.1	0.819
**Ceramides**	Cer(d18:2/22:0)	7.5±1.1	6.9±0.4	0.382	8.4±2.3	8.1±1.4	0.885
**Ceramides**	Cer(d18:2/23:0)	0.8±0.2	0.7±0.1	0.52	1±0.3	1±0.1	0.764
**Ceramides**	Cer(d18:2/24:0)	20.9±2.2	21±2.6	0.951	20.8±4.7	22±1.2	0.68
**Ceramides**	Cer(d18:2/24:1)	5.6±0.9	5.6±0.4	0.901	7.5±2	6.3±0.7	0.331
**Cholesteryl esters**	CE(14:0)	2.5±1.5	3.7±1.3	0.345	5.8±3.7	6.1±3.4	0.924
**Cholesteryl esters**	CE(15:1)	1.9±1.1	2±0.9	0.892	2.7±2.5	4.2±0.9	0.361
**Cholesteryl esters**	CE(16:1)	1.7±0.6	3±0.9	0.066	1.8±1.3	0.9±0.5	0.307
**Cholesteryl esters**	CE(18:1)	3.2±1.1	4.4±0.4	0.119	8.1±5	4.5±1	0.269
**Cholesteryl esters**	CE(18:2)	28.4±16.7	53±21.1	0.164	54.4±30.6	55.2±33.4	0.979

**Cholesteryl esters**	CE(18:3)	1.2±0.7	2.1±1.3	0.322	1.6±1.1	1.6±0.7	0.943
**Cholesteryl esters**	CE(20:3)	1±0.5	1.6±1.1	0.343	1.7±0.9	1.3±0.5	0.472
**Cholesteryl esters**	CE(20:4)	36.4±22.2	57.7±19.6	0.258	41.2±20.5	56.5±30.4	0.497
**Cholesteryl esters**	CE(20:5)	2±0.7	2.9±1.1	0.278	1.9±1.2	2.2±0.4	0.656
**Cholesteryl esters**	CE(22:5)	0.6±0.3	0.9±0.2	0.155	1.1±0.4	1±0.5	0.878
**Cholesteryl esters**	CE(22:6)	9.3±4	12.5±3.6	0.339	12.6±7.3	14.8±6.3	0.711
**Diacylglyceroles**	DG(14:0_18:1)	4.2±0.6	5.2±1.3	0.239	6.1±3.4	6.6±3.1	0.867
**Diacylglyceroles**	DG(14:0_18:2)	4.4±1.1	5.3±1.4	0.386	8.5±5.8	9.1±4.2	0.886
**Diacylglyceroles**	DG(14:1_18:1)	0.3±0.2	0.6±0.1	0.011*§	0.4±0.2	0.6±0.3	0.248
**Diacylglyceroles**	DG(16:0_16:1)	4.4±0.7	4.9±0.4	0.272	3.9±2.5	3.7±1.2	0.917
**Diacylglyceroles**	DG(16:0_18:1)	19.2±2.8	24.2±7.1	0.298	26.5±14.5	26.2±7	0.97
**Diacylglyceroles**	DG(16:0_18:2)	15.5±2.3	19.8±6.6	0.33	25.3±16.8	24.8±7.5	0.964
**Diacylglyceroles**	DG(16:0_20:3)	0.4±0.1	0.4±0.1	0.411	0.5±0.2	0.5±0.2	0.836
**Diacylglyceroles**	DG(16:0_20:4)	1.2±0.3	1.3±0.2	0.653	1.2±0.4	1.6±0.4	0.179
**Diacylglyceroles**	DG(16:1_18:0)	0.4±0.2	0.5±0.2	0.268	0.3±0.3	0.3±0.2	0.625
**Diacylglyceroles**	DG(16:1_18:2)	3.5±0.4	4.5±0.8	0.081	4.3±3.1	3.3±1.5	0.628
**Diacylglyceroles**	DG(17:0_18:1)	4±0.7	5±1.4	0.271	4.6±1.2	5.2±0.7	0.513
**Diacylglyceroles**	DG(18:0_20:4)	4.7±0.7	6.4±1	0.043*§	5.2±0.8	6.5±0.7	0.062
**Diacylglyceroles**	DG(18:1_18:1)	5.8±0.5	8.4±1.2	0.009*§	8±4.1	7.5±3.4	0.866
**Diacylglyceroles**	DG(18:1_18:2)	17.6±1.4	24.9±3	0.009*§	34.6±23.7	29.2±12.2	0.741
**Diacylglyceroles**	DG(18:1_18:3)	1.4±0.5	2.2±0.6	0.125	2.1±1.5	1.9±0.8	0.838
**Diacylglyceroles**	DG(18:1_20:0)	0.7±0.1	0.8±0.2	0.137	0.8±0.2	0.9±0.5	0.857
**Diacylglyceroles**	DG(18:1_20:1)	0.2±0.1	0.2±0.1	0.218	0.3±0.2	0.2±0.1	0.683
**Diacylglyceroles**	DG(18:1_20:2)	0.2±0.1	0.2±0.1	0.871	0.3±0.2	0.3±0.1	0.943
**Diacylglyceroles**	DG(18:1_20:4)	1.5±0.2	1.7±0.2	0.208	1.8±0.2	1.7±0.5	0.94
**Diacylglyceroles**	DG(18:2_18:2)	15.9±2.8	18.5±2	0.225	44.3±38.5	32±12.7	0.619
**Diacylglyceroles**	DG(18:2_20:4)	0.8±0.1	1±0.1	0.035*§	1.1±0.5	1.3±0.4	0.456
**Dihydroceramides**	Cer(d18:0/18:0(OH))	4.7±3.3	7.7±1.2	0.18	4.3±3.9	4.7±1.5	0.882
**Dihydroceramides**	Cer(d18:0/22:0)	0.6±0.1	0.7±0.2	0.484	0.5±0.2	0.7±0.1	0.048*
**Dihydroceramides**	Cer(d18:0/24:0)	0.9±0.3	1.1±0.2	0.113	0.7±0.2	1±0.3	0.073
**Dihydroceramides**	Cer(d18:0/24:1)	0.5±0.1	0.6±0.2	0.322	0.5±0.1	0.5±0.2	0.934
**Fatty acids**	Arachidonic acid	363.8±101.5	356.5±116.2	0.938	374.5±168.4	396.3±157.8	0.876
**Fatty acids**	Docosahexaenoic acid	211.5±61.6	185±65.9	0.629	223.3±122	281±87.3	0.53
**Fatty acids**	Eicosapentaenoic acid	9.3±3.7	9.1±3	0.928	13.3±7.4	16.2±7.9	0.655
**Fatty acids**	Elaidic acid	94.3±19.9	107.1±29.6	0.555	204.5±86	127.5±48.9	0.226
**Fatty acids**	Linoleic acid	232.8±97.7	286.5±44.8	0.42	524±277.6	476.3±170.7	0.809
**Fatty acids**	Eicosadienoic acid	3.2±0.7	3.1±0.4	0.852	7.3±3.7	5.5±1.7	0.47
**Fatty acids**	Dihomo-gamma-linolenic acid	14.2±4.4	16.3±2	0.468	21.7±5.3	18.1±5.7	0.449
**Lysophosphatidylcholines**	lysoPC a C14:0	4.4±0.9	4.9±1.2	0.527	9.5±5.2	10.2±4.2	0.856

**Lysophosphatidylcholines**	lysoPC a C16:0	158±13.4	170±15.8	0.354	191.8±58.2	218.8±39.3	0.53
**Lysophosphatidylcholines**	lysoPC a C16:1	3±0.5	3.4±0.4	0.258	4.1±2.5	3.2±1.3	0.609
**Lysophosphatidylcholines**	lysoPC a C17:0	2±0.4	2±0.3	0.819	1.9±0.2	2.2±0.3	0.078
**Lysophosphatidylcholines**	lysoPC a C18:0	131.5±6.3	139.5±7.5	0.205	99±10.1	136.3±20.9	0.032*
**Lysophosphatidylcholines**	lysoPC a C18:1	32.9±5.4	34.5±6.9	0.77	71.1±43.2	69±34	0.949
**Lysophosphatidylcholines**	lysoPC a C18:2	43.1±9.5	48.6±16.5	0.63	150.6±131	146±73.3	0.96
**Lysophosphatidylcholines**	lysoPC a C20:3	4.8±1.3	5±2.2	0.899	14±10.9	10.7±6.5	0.664
**Lysophosphatidylcholines**	lysoPC a C20:4	66.2±13.5	58.3±19.8	0.591	128.3±73.4	137.7±76.6	0.884
**Lysophosphatidylcholines**	lysoPC a C24:0	1.7±0.2	2±0.3	0.077	2±0.5	1.6±0.4	0.265
**Lysophosphatidylcholines**	lysoPC a C26:0	2.1±0.6	2±0.4	0.755	2.8±1.2	2.4±0.5	0.615
**Lysophosphatidylcholines**	lysoPC a C26:1	1.1±0.2	0.9±0.1	0.074	1±0.3	0.9±0.1	0.378
**Lysophosphatidylcholines**	lysoPC a C28:0	17.1±1.8	14.4±2.4	0.161	34.7±5.7	37.2±10.4	0.723
**Lysophosphatidylcholines**	lysoPC a C28:1	2.6±0.3	2.9±0.5	0.383	2.8±0.7	2.8±0.3	0.967
**Phosphatidylcholines**	PC aa C24:0	0.7±0.1	0.8±0.1	0.117	0.8±0.3	1.1±0.2	0.244
**Phosphatidylcholines**	PC aa C26:0	4.1±0.8	4.5±0.6	0.453	5±1.1	6±0.9	0.222
**Phosphatidylcholines**	PC aa C28:1	13.2±1.3	12.8±0.7	0.644	12.8±1.8	15±1.6	0.162
**Phosphatidylcholines**	PC aa C30:0	169±14.2	187.3±23.9	0.299	199.8±14.2	220±24.9	0.266
**Phosphatidylcholines**	PC aa C30:2	0.6±0.3	0.4±0.2	0.519	0.8±0.2	0.5±0.2	0.122
**Phosphatidylcholines**	PC aa C32:0	1917.3±102.8	2042.5±151.3	0.281	1791.8±265.8	2027.5±108.7	0.205
**Phosphatidylcholines**	PC aa C32:1	235.8±24.7	269±36.5	0.239	178.3±14.7	169.3±26.9	0.629
**Phosphatidylcholines**	PC aa C32:2	52.9±6.2	63.6±7.8	0.112	67.8±3.1	80.7±15.9	0.216
**Phosphatidylcholines**	PC aa C32:3	4±0.5	4.8±0.5	0.084	4.6±0.7	5.3±1.2	0.394
**Phosphatidylcholines**	PC aa C34:1	1488.8±81.1	1624.3±36.5	0.039*	1314.5±105.1	1438.3±139.6	0.266
**Phosphatidylcholines**	PC aa C34:2	1290.3±124	1420.8±150.6	0.291	1645.5±240.8	1823±187.8	0.353
**Phosphatidylcholines**	PC aa C34:3	69.8±6.9	80.1±8.8	0.158	62.2±1.3	68±10.9	0.395
**Phosphatidylcholines**	PC aa C34:4	28.8±3.2	33.1±3.2	0.153	31.1±2.8	35±5.8	0.333
**Phosphatidylcholines**	PC aa C36:0	62±1.5	62±5.6	0.995	56.3±10.2	57.6±5.5	0.855
**Phosphatidylcholines**	PC aa C36:1	514±51.6	568.5±27	0.156	449.8±53.1	541.3±66.6	0.112
**Phosphatidylcholines**	PC aa C36:2	967.5±110	1078.8±99.7	0.242	1044.8±51.1	1234.3±138.8	0.069
**Phosphatidylcholines**	PC aa C36:3	432.5±50.7	485.5±31.7	0.176	510±19.8	555.5±62.8	0.277
**Phosphatidylcholines**	PC aa C36:4	2069.8±130.2	2191±152.4	0.336	1636±184.1	2019.3±149.5	0.032*
**Phosphatidylcholines**	PC aa C36:5	69.3±4.2	71.5±3	0.468	50.7±6.7	63.5±6.6	0.056
**Phosphatidylcholines**	PC aa C36:6	47.1±5	51.1±1.5	0.226	49.5±11.1	59.6±7.7	0.242
**Phosphatidylcholines**	PC aa C38:0	334.8±30	322.8±8.6	0.529	275.3±48.2	252.3±55.1	0.606
**Phosphatidylcholines**	PC aa C38:1	121±11.4	116.3±3.6	0.515	95.9±17.7	98.3±17.9	0.876
**Phosphatidylcholines**	PC aa C38:3	154.5±16.7	173.5±27.7	0.348	142.3±19.8	145.8±13.5	0.808
**Phosphatidylcholines**	PC aa C38:4	1550.8±149.1	1626.8±88.1	0.476	1085±151.8	1365±185.1	0.09
**Phosphatidylcholines**	PC aa C38:5	559±46	614.8±15.5	0.094	433±62.7	498±60.1	0.243
**Phosphatidylcholines**	PC aa C38:6	2087.5±78.9	2169.3±142.8	0.419	1652.8±417.8	2370.8±162.1	0.033*

**Phosphatidylcholines**	PC aa C40:1	3.5±0.3	4.2±1.2	0.347	3.8±0.6	3.2±0.3	0.121
**Phosphatidylcholines**	PC aa C40:2	4.3±0.5	6.4±2	0.113	5.3±0.7	4.7±0.7	0.275
**Phosphatidylcholines**	PC aa C40:3	4.9±0.4	5.1±0.5	0.46	6.1±0.8	5.1±0.7	0.117
**Phosphatidylcholines**	PC aa C40:4	26.3±3.6	31.1±1.8	0.079	27.2±3.3	29.4±3.4	0.448
**Phosphatidylcholines**	PC aa C40:5	55.5±5.9	60.8±1.3	0.172	47.1±9.1	50.1±6.4	0.658
**Phosphatidylcholines**	PC aa C40:6	398±6.8	426.8±21.1	0.065	271±40.6	368±39.2	0.025*
**Phosphatidylcholines**	PC aa C42:0	1±0.2	1.2±0.2	0.128	0.8±0.3	0.7±0.3	0.466
**Phosphatidylcholines**	PC aa C42:1	1.4±0.2	1.5±0.3	0.306	1.1±0.2	1.2±0.3	0.621
**Phosphatidylcholines**	PC aa C42:2	1.8±0.3	1.9±0.1	0.408	1.9±0.2	1.6±0.3	0.165
**Phosphatidylcholines**	PC aa C42:4	4.6±0.7	6.7±0.8	0.01*	4.6±1.2	4.1±0.3	0.532
**Phosphatidylcholines**	PC aa C42:5	7.2±0.4	7.7±0.2	0.066	5.6±0.8	5.7±1.1	0.938
**Phosphatidylcholines**	PC aa C42:6	4.4±0.3	4.9±0.6	0.221	3.9±0.3	4.4±0.6	0.188
**Phosphatidylcholines**	PC ae C30:0	32.3±3.2	29.5±3.5	0.336	54±11.6	54.5±5.4	0.943
**Phosphatidylcholines**	PC ae C30:1	2.3±0.3	2.3±0.2	0.959	3±0.4	2.6±0.4	0.231
**Phosphatidylcholines**	PC ae C30:2	0.7±0.1	0.6±0.1	0.401	0.6±0.2	0.6±0.1	0.661
**Phosphatidylcholines**	PC ae C32:1	46.9±3.1	48.7±1.7	0.387	52.2±7.8	41.3±5.1	0.088
**Phosphatidylcholines**	PC ae C32:2	11.8±0.7	11.7±0.7	0.86	24.6±8.8	20.8±3.5	0.506
**Phosphatidylcholines**	PC ae C34:0	21.7±1.6	22.9±2.6	0.525	20.4±2.1	21.1±2.9	0.754
**Phosphatidylcholines**	PC ae C34:1	157±9.1	163.5±2.3	0.273	164.3±32.8	134.8±31.7	0.305
**Phosphatidylcholines**	PC ae C34:2	73.7±5.6	76.7±2.9	0.443	104.1±25.5	93.2±7.5	0.501
**Phosphatidylcholines**	PC ae C34:3	20.8±1.8	21.3±1.7	0.76	30.8±4.2	31.1±5.7	0.949
**Phosphatidylcholines**	PC ae C36:0	8.8±1.5	7.6±1.3	0.303	7.5±1.6	7.7±1.2	0.914
**Phosphatidylcholines**	PC ae C36:1	25.6±4.2	27.7±2.7	0.48	23.9±4.2	24.8±3.5	0.799
**Phosphatidylcholines**	PC ae C36:2	25.3±4.2	26.6±2.7	0.662	31.2±3.9	29.4±3.5	0.584
**Phosphatidylcholines**	PC ae C36:3	25.7±2.5	24.8±1.4	0.59	33.1±7.4	26.9±2.5	0.213
**Phosphatidylcholines**	PC ae C36:4	245±5.7	246.5±12.9	0.86	297.5±51.9	293.3±27.7	0.905
**Phosphatidylcholines**	PC ae C36:5	227.5±11.8	239±10.1	0.244	235±25.9	258.3±36.2	0.401
**Phosphatidylcholines**	PC ae C38:0	56.2±1.5	60.9±2.1	0.02*	37.5±5.6	46.5±4.6	0.075
**Phosphatidylcholines**	PC ae C38:1	3.2±0.7	4.6±1.4	0.16	3.6±0.6	3.6±1.8	0.981
**Phosphatidylcholines**	PC ae C38:2	6.4±1.2	7±1.8	0.605	7.2±1.2	8.4±1.9	0.388
**Phosphatidylcholines**	PC ae C38:3	7±0.4	7.4±1.3	0.644	6.7±1	8.5±3	0.354
**Phosphatidylcholines**	PC ae C38:4	48.5±6.4	49.1±3	0.877	51.7±8.4	50.3±7.6	0.839
**Phosphatidylcholines**	PC ae C38:5	136.3±14.2	140.8±16.2	0.729	191.3±46.6	156.5±17.1	0.271
**Phosphatidylcholines**	PC ae C38:6	1151.3±61	1120.8±62.7	0.568	1451.5±374.5	1606.5±37.4	0.503
**Phosphatidylcholines**	PC ae C40:1	99.4±6.4	104.9±16.4	0.607	82.1±15.2	108.1±8.9	0.043*
**Phosphatidylcholines**	PC ae C40:2	5.1±0.3	5.9±0.9	0.203	5±1.2	5.8±0.7	0.301
**Phosphatidylcholines**	PC ae C40:3	4.7±1.4	4.3±0.7	0.654	3.5±1.1	3.4±1.4	0.953
**Phosphatidylcholines**	PC ae C40:4	9.9±0.7	11.1±1.1	0.14	8.8±1.6	9.3±2.1	0.748
**Phosphatidylcholines**	PC ae C40:5	10.9±1.4	10.4±0.7	0.608	12.8±1.5	12.2±2.2	0.717

**Phosphatidylcholines**	PC ae C40:6	69.6±2.6	65.9±4.3	0.254	84.1±15.4	87.6±11.5	0.764
**Phosphatidylcholines**	PC ae C42:0	5.8±0.3	6.5±0.7	0.134	4.7±0.5	4.7±0.4	0.995
**Phosphatidylcholines**	PC ae C42:1	7.9±0.8	8.7±1.2	0.359	7.1±0.7	7.3±1.3	0.857
**Phosphatidylcholines**	PC ae C42:2	5.8±0.3	6.4±0.8	0.249	4.7±1.2	5.1±0.8	0.643
**Phosphatidylcholines**	PC ae C42:3	25.6±3.3	26.8±2.5	0.608	15.7±5.9	20.9±0.9	0.18
**Phosphatidylcholines**	PC ae C42:4	1.8±0.3	1.8±0.3	0.999	1.6±0.4	1.7±0.4	0.681
**Phosphatidylcholines**	PC ae C42:5	3.5±0.3	4.6±1	0.102	3.3±0.5	2.9±0.3	0.217
**Phosphatidylcholines**	PC ae C44:3	1.7±0.3	1.6±0.2	0.53	1.3±0.3	1.5±0.2	0.216
**Phosphatidylcholines**	PC ae C44:4	1.3±0.2	1.2±0.2	0.842	1±0.3	1.2±0.1	0.262
**Phosphatidylcholines**	PC ae C44:5	2.3±0.5	2.2±0.1	0.766	1.8±0.7	2.3±0.1	0.19
**Phosphatidylcholines**	PC ae C44:6	1.2±0.5	1±0.2	0.403	0.9±0.3	0.8±0.1	0.296
**Dihexosylceramides**	Hex2Cer(d18:1/14:0)	2±0.3	2.4±0.7	0.345	2.5±0.3	2.6±1	0.791
**Dihexosylceramides**	Hex2Cer(d18:1/16:0)	3.6±0.5	3.9±0.8	0.543	4.2±0.3	4.3±1.2	0.921
**Dihexosylceramides**	Hex2Cer(d18:1/18:0)	2.7±0.4	3.3±0.7	0.278	3.1±0.3	2.9±0.8	0.676
**Dihexosylceramides**	Hex2Cer(d18:1/20:0)	0.3±0.1	0.4±0.1	0.351	0.4±0.1	0.4±0.1	0.684
**Dihexosylceramides**	Hex2Cer(d18:1/22:0)	0.9±0.2	0.9±0.1	0.948	0.9±0.2	1±0.2	0.22
**Dihexosylceramides**	Hex2Cer(d18:1/24:0)	0.8±0.1	0.9±0.1	0.292	0.9±0.1	1±0.2	0.336
**Dihexosylceramides**	Hex2Cer(d18:1/24:1)	0.3±0.1	0.4±0.1	0.166	0.5±0.1	0.5±0.1	0.899
**Dihexosylceramides**	Hex2Cer(d18:1/26:0)	0.1±0.1	0.1±0.1	0.856	0.1±0.1	0.1±0.1	0.027*§
**Dihexosylceramides**	Hex2Cer(d18:1/26:1)	0.5±0.1	0.5±0.2	0.659	0.5±0.1	0.4±0.1	0.362
**Trihexosylceramides**	Hex3Cer(d18:1/16:0)	2±0.4	2.3±0.2	0.121	5.1±2	4.2±0.8	0.5
**Trihexosylceramides**	Hex3Cer(d18:1/18:0)	0.9±0.2	0.9±0.2	0.957	1.2±0.3	1±0.3	0.493
**Trihexosylceramides**	Hex3Cer(d18:1/24:1)	1.3±0.3	1.5±0.3	0.219	2.9±1	2.5±0.8	0.616
**Trihexosylceramides**	Hex3Cer(d18:1/26:1)	1.7±0.5	1.9±0.4	0.654	1.9±0.5	1.9±0.2	0.941
**Trihexosylceramides**	Hex3Cer(d18:1_20:0)	1.4±0.2	1.4±0.2	0.497	2.9±0.4	3.7±1.6	0.457
**Trihexosylceramides**	Hex3Cer(d18:1_22:0)	3.8±0.5	4.2±0.7	0.338	6.2±1	7.2±1.8	0.439
**Hexosylceramides**	HexCer(d16:1/22:0)	0.1±0.1	0.1±0.1	0.205	0.1±0.1	0.1±0.1	0.688
**Hexosylceramides**	HexCer(d18:1/14:0)	0.2±0.1	0.2±0.1	0.591	0.2±0.1	0.2±0.1	0.081
**Hexosylceramides**	HexCer(d18:1/16:0)	3.8±0.9	4.6±0.6	0.244	7±2.8	5.7±0.7	0.457
**Hexosylceramides**	HexCer(d18:1/18:0)	0.9±0.3	1.1±0.1	0.292	1.4±0.4	1.4±0.4	0.841
**Hexosylceramides**	HexCer(d18:1/18:1)	0.4±0.1	0.5±0.1	0.029*	0.5±0.2	0.5±0.2	0.903
**Hexosylceramides**	HexCer(d18:1/20:0)	3.2±0.8	3.7±0.3	0.257	5±1	5.5±1.3	0.603
**Hexosylceramides**	HexCer(d18:1/22:0)	16±3.2	18.6±1.2	0.225	23.7±5.7	24.3±4.6	0.891
**Hexosylceramides**	HexCer(d18:1/23:0)	3.5±0.8	3.8±0.2	0.531	5.5±1.9	5.3±1.1	0.852
**Hexosylceramides**	HexCer(d18:1/24:0)	7.2±1.2	8.9±1.1	0.116	9.8±1.2	10.6±1.9	0.569
**Hexosylceramides**	HexCer(d18:1/24:1)	13.8±1.6	17.5±2.4	0.064	19.1±4.4	18.9±3.2	0.945
**Hexosylceramides**	HexCer(d18:1/26:0)	0.3±0.1	0.4±0.1	0.238	0.4±0.2	0.5±0.1	0.433
**Hexosylceramides**	HexCer(d18:1/26:1)	0.9±0.2	1.1±0.2	0.056	1.1±0.2	1.2±0.5	0.614
**Hexosylceramides**	HexCer(d18:2/16:0)	0.2±0.1	0.2±0.1	0.999	0.4±0.1	0.4±0.1	0.786

**Hexosylceramides**	HexCer(d18:2/20:0)	0.2±0.1	0.2±0.1	0.28	0.2±0.1	0.2±0.1	0.701
**Hexosylceramides**	HexCer(d18:2/22:0)	1.4±0.2	1.7±0.1	0.045*	2.1±0.5	2.3±0.3	0.67
**Hexosylceramides**	HexCer(d18:2/23:0)	0.7±0.1	0.7±0.1	0.77	0.8±0.2	0.9±0.2	0.446
**Hexosylceramides**	HexCer(d18:2/24:0)	3.7±0.8	4.4±1.2	0.416	4.9±0.6	5.3±1.2	0.613
**Indoles and derivatives**	Indoleacetic acid	1±0.2	1±0.2	0.999	1.1±0.4	1.3±0.3	0.515
**Indoles and derivatives**	Indole-3-propionic acid	0.3±0.2	0.6±0.2	0.101	0.4±0.1	0.4±0.2	0.823
**Indoles and derivatives**	Indoxyl sulfate	0.6±0.4	1.4±0.2	0.011*§	6.8±7.8	1.4±0.6	0.28
**Sphingomyelins**	SM (OH) C14:1	23.6±2.5	22.9±2.9	0.744	16.5±2.4	19.9±3.6	0.208
**Sphingomyelins**	SM (OH) C16:1	6.9±0.9	7.1±1	0.796	6.8±0.5	6.8±1.1	0.983
**Sphingomyelins**	SM (OH) C22:1	39.3±5.8	43.1±3.6	0.372	36.2±4.4	48.3±6.8	0.04*
**Sphingomyelins**	SM (OH) C22:2	12.4±1.8	12.8±1.9	0.805	11.1±1.2	14.1±1.8	0.05*
**Sphingomyelins**	SM (OH) C24:1	4.2±0.6	4.9±0.8	0.228	4±0.9	5.2±0.9	0.106
**Sphingomyelins**	SM C16:0	1207.8±85.5	1225.8±36	0.749	1076.8±146.9	1272.8±104.6	0.109
**Sphingomyelins**	SM C16:1	199.5±6.4	201±11.1	0.846	210±42.1	226±22.6	0.583
**Sphingomyelins**	SM C18:0	63.6±8.4	72.1±3.9	0.16	57.4±2.7	70.1±9.4	0.065
**Sphingomyelins**	SM C18:1	10.8±0.7	11.1±0.5	0.522	8.3±0.5	10.4±1.2	0.025*
**Sphingomyelins**	SM C20:2	b.dl.	b.dl.	n.a.	b.dl.	b.dl.	n.a.
**Sphingomyelins**	SM C22:3	0.7±1.2	1.5±1.7	0.527	1.9±1.2	2.5±1.9	0.633
**Sphingomyelins**	SM C24:0	212.5±21.3	230±21.2	0.352	175.3±23.9	246.3±46.8	0.058
**Sphingomyelins**	SM C24:1	280.8±34.4	298.5±40.4	0.583	249.3±28	292.8±50.5	0.24
**Sphingomyelins**	SM C26:0	1.4±0.5	1.6±0.5	0.47	1.2±0.4	1.5±0.4	0.382
**Sphingomyelins**	SM C26:1	1.3±0.1	1.7±0.8	0.441	1.1±0.5	1.5±0.4	0.253
**Triacylglycerole**	TG(14:0_32:2)	66.5±29	155.3±103.2	0.202	132.3±139.8	137±92.7	0.963
**Triacylglycerole**	TG(14:0_34:0)	12±3.9	29.8±22.5	0.227	19.4±17.9	19.8±11.6	0.977
**Triacylglycerole**	TG(14:0_34:1)	89.5±33.8	228.8±170.6	0.215	121±117.5	114.6±81.1	0.941
**Triacylglycerole**	TG(14:0_34:2)	99.5±38.3	233.6±146.8	0.177	158.2±170.5	137.8±86	0.86
**Triacylglycerole**	TG(14:0_34:3)	27.2±10.9	57±22.4	0.084	30.8±31	28±15.4	0.894
**Triacylglycerole**	TG(14:0_35:1)	1.1±0.4	2.4±1.8	0.266	1.7±1.1	1.6±0.9	0.912
**Triacylglycerole**	TG(14:0_35:2)	1.7±0.7	3.4±2.2	0.214	3.3±2.8	2.8±1.2	0.75
**Triacylglycerole**	TG(14:0_36:1)	13±5.1	38.9±36.6	0.27	15.2±11	24.2±21.6	0.542
**Triacylglycerole**	TG(14:0_36:2)	67.5±28	178.4±136.8	0.219	61±43.5	88.9±81.1	0.619
**Triacylglycerole**	TG(14:0_36:3)	68±29.1	165.8±123.5	0.231	90±82.6	107±85.6	0.813
**Triacylglycerole**	TG(14:0_36:4)	20.3±7.3	42.5±26.9	0.217	52.6±58.5	47.3±26	0.891
**Triacylglycerole**	TG(14:0_38:4)	4±1.2	5.5±1.7	0.247	8.1±7.1	8.7±4	0.895
**Triacylglycerole**	TG(14:0_38:5)	3.7±1.3	5±1.7	0.325	9.2±8.4	9.1±4.5	0.986
**Triacylglycerole**	TG(16:0_28:1)	48.9±21.9	108.7±43.1	0.076	17.7±7.5	30.1±23.9	0.422
**Triacylglycerole**	TG(16:0_28:2)	9±4.3	19.1±10.5	0.173	5.9±2.7	9.8±8.9	0.491
**Triacylglycerole**	TG(16:0_30:2)	132.1±61.5	312.5±241.4	0.257	103.2±61.1	188.7±173.6	0.452
**Triacylglycerole**	TG(16:0_32:0)	24.5±7.4	59.4±38.6	0.175	48.1±51.5	38.3±17.2	0.764

**Triacylglycerole**	TG(16:0_32:1)	110.6±43.3	267.5±176.5	0.186	145.4±144.4	127.9±86.3	0.863
**Triacylglycerole**	TG(16:0_32:2)	81.8±32.4	191.8±130.3	0.206	160.8±180.5	129.3±74.1	0.79
**Triacylglycerole**	TG(16:0_32:3)	9.1±3.4	17.8±7.2	0.105	14.1±13.6	12.7±6.4	0.877
**Triacylglycerole**	TG(16:0_33:1)	1.9±0.8	4±1.7	0.091	5.4±5.4	3.1±1.2	0.493
**Triacylglycerole**	TG(16:0_33:2)	1.4±0.7	2.6±0.8	0.09	4.8±5	2.5±1	0.465
**Triacylglycerole**	TG(16:0_34:0)	10±3.2	24.7±17.6	0.204	17.8±18.2	12.2±4.5	0.623
**Triacylglycerole**	TG(16:0_34:1)	99.3±30	260.7±191.9	0.201	162.7±173.4	111.7±61.1	0.649
**Triacylglycerole**	TG(16:0_34:2)	120.3±40.5	291.5±179.4	0.158	213.8±249.1	132.4±63.9	0.603
**Triacylglycerole**	TG(16:0_34:3)	38±13.5	78.7±32.4	0.091	42.2±45.8	30.1±14.5	0.677
**Triacylglycerole**	TG(16:0_34:4)	4.7±1.6	7.4±1.2	0.055	10±9.7	7.9±3.5	0.742
**Triacylglycerole**	TG(16:0_35:1)	1±0.5	2.4±1.9	0.242	2.2±1.8	1.5±0.6	0.497
**Triacylglycerole**	TG(16:0_35:2)	1.7±0.6	4.1±2.1	0.107	3.8±3.3	2.2±0.7	0.456
**Triacylglycerole**	TG(16:0_35:3)	1.2±0.4	2.1±1.1	0.209	3.1±3.4	1.6±0.6	0.495
**Triacylglycerole**	TG(16:0_36:2)	91±29.1	233.5±164.4	0.19	96.3±81.8	91.4±64.3	0.938
**Triacylglycerole**	TG(16:0_36:3)	95.6±32	231.9±166.6	0.214	151.7±157.6	121.5±74.4	0.775
**Triacylglycerole**	TG(16:0_36:4)	31.2±8.6	62±37.6	0.216	91.4±109.4	55.5±25	0.6
**Triacylglycerole**	TG(16:0_36:5)	4.5±1.1	6.9±1.6	0.073	13.6±14.7	9.9±4.4	0.692
**Triacylglycerole**	TG(16:0_36:6)	3.8±1.4	4.2±1.4	0.718	12.8±12.2	11.4±5.4	0.864
**Triacylglycerole**	TG(16:0_37:3)	0.4±0.2	0.8±0.5	0.258	0.7±0.7	0.5±0.2	0.539
**Triacylglycerole**	TG(16:0_38:1)	0.8±0.3	2.2±1.8	0.205	1.3±1	1.3±0.5	0.933
**Triacylglycerole**	TG(16:0_38:2)	2.6±0.8	6.3±5.5	0.291	3.4±2.9	3.2±1.4	0.93
**Triacylglycerole**	TG(16:0_38:3)	3.1±0.7	6.3±4.5	0.248	6.8±7.1	5.1±1.8	0.705
**Triacylglycerole**	TG(16:0_38:4)	4.8±0.9	7.2±1.6	0.06	11.9±12	8.5±4.1	0.653
**Triacylglycerole**	TG(16:0_38:5)	6.4±1.6	8±1	0.193	13.8±13	11.6±5.1	0.797
**Triacylglycerole**	TG(16:0_38:6)	7.3±1.6	8±1.5	0.601	20±18.5	16.5±7.8	0.768
**Triacylglycerole**	TG(16:0_38:7)	1.4±0.4	1.7±0.7	0.515	3.3±3.6	2.6±1.4	0.728
**Triacylglycerole**	TG(16:0_40:6)	2.5±0.5	2.6±0.3	0.822	7.8±7.5	5.7±2.5	0.671
**Triacylglycerole**	TG(16:0_40:7)	3.5±0.8	3.4±1.1	0.874	14.4±15.1	9±4.7	0.575
**Triacylglycerole**	TG(16:0_40:8)	2.8±0.7	2.7±1	0.826	11.1±11.7	8.3±4.4	0.71
**Triacylglycerole**	TG(16:1_28:0)	54.1±26.5	106.7±31.9	0.07	28.6±19	34.1±21.3	0.752
**Triacylglycerole**	TG(16:1_30:1)	83±38.4	178.5±66	0.074	20.6±11.5	42.3±39.4	0.396
**Triacylglycerole**	TG(16:1_32:0)	16.7±6.2	35.8±12.1	0.05	15.5±16.4	9.7±5.3	0.577
**Triacylglycerole**	TG(16:1_32:1)	51.7±22.1	119.7±41.7	0.047*§	24.2±19	23.9±16.9	0.985
**Triacylglycerole**	TG(16:1_32:2)	30.1±12.8	67.6±24.7	0.058	21±20.4	20.2±13.7	0.956
**Triacylglycerole**	TG(16:1_33:1)	0.7±0.3	2.4±0.7	0.008*§	1.2±0.8	0.6±0.6	0.372
**Triacylglycerole**	TG(16:1_34:0)	6.3±1.9	13.9±5.2	0.055	4.5±4.1	3.7±2.2	0.76
**Triacylglycerole**	TG(16:1_34:1)	52.4±19.6	121.6±49.5	0.066	28.1±25.2	22.9±15.6	0.77
**Triacylglycerole**	TG(16:1_34:2)	53.9±22	124.3±44.8	0.051	30.3±30.3	24.2±15.6	0.766
**Triacylglycerole**	TG(16:1_34:3)	13.9±5.9	31.2±10.5	0.047*§	6.2±5.6	5.3±3.5	0.826

**Triacylglycerole**	TG(16:1_36:1)	6.9±2.8	16.9±9.3	0.122	3.1±2.4	4.5±3.5	0.591
**Triacylglycerole**	TG(16:1_36:2)	42±17.6	98.6±40.8	0.07	16.2±11.6	20.8±16.5	0.705
**Triacylglycerole**	TG(16:1_36:3)	36.6±15.6	81.2±34.4	0.087	23.1±20.3	21±15.5	0.893
**Triacylglycerole**	TG(16:1_36:4)	8.9±3.3	17.7±5.9	0.062	11.8±13.2	7.3±3.6	0.592
**Triacylglycerole**	TG(16:1_36:5)	1.2±0.4	2.2±0.5	0.021*§	1.7±1.8	1.1±0.5	0.579
**Triacylglycerole**	TG(16:1_38:3)	1.1±0.4	2.4±1.1	0.092	1.3±1.1	1±0.5	0.684
**Triacylglycerole**	TG(16:1_38:4)	1.7±0.3	2.5±0.4	0.013*§	1.9±1.6	1.7±0.4	0.873
**Triacylglycerole**	TG(16:1_38:5)	1.6±0.4	2.3±0.6	0.104	2.2±1.8	1.5±0.5	0.553
**Triacylglycerole**	TG(17:0_32:1)	0.7±0.2	2.1±2	0.247	1±1	1.1±0.5	0.792
**Triacylglycerole**	TG(17:0_34:1)	1±0.4	1.7±1.4	0.375	1.1±1	1.1±0.7	0.955
**Triacylglycerole**	TG(17:0_34:2)	0.9±0.4	2.1±1.6	0.257	1.2±1.3	1.1±0.4	0.852
**Triacylglycerole**	TG(17:0_34:3)	0.3±0.2	0.7±0.4	0.124	0.4±0.3	0.3±0.3	0.925
**Triacylglycerole**	TG(17:0_36:3)	0.6±0.3	1.5±1.5	0.339	1.1±1	0.9±0.8	0.851
**Triacylglycerole**	TG(17:0_36:4)	0.3±0.1	0.6±0.4	0.271	0.8±0.7	0.5±0.2	0.556
**Triacylglycerole**	TG(17:1_32:1)	1.3±0.7	2.6±1.3	0.156	1.4±1.3	1.3±0.7	0.874
**Triacylglycerole**	TG(17:1_34:1)	1.1±0.4	2.7±1.4	0.109	1.4±1.2	0.9±0.6	0.612
**Triacylglycerole**	TG(17:1_34:2)	1.5±0.6	2.8±1.6	0.214	1.4±1.4	1.2±0.6	0.851
**Triacylglycerole**	TG(17:1_34:3)	0.5±0.2	1±0.4	0.107	0.4±0.3	0.5±0.2	0.597
**Triacylglycerole**	TG(17:1_36:3)	0.8±0.4	1.9±1.3	0.199	1±0.8	0.9±0.5	0.927
**Triacylglycerole**	TG(17:1_36:4)	0.4±0.1	0.7±0.4	0.266	0.8±0.6	0.5±0.2	0.533
**Triacylglycerole**	TG(17:1_38:5)	0.4±0.1	0.5±0.1	0.063	0.5±0.4	0.5±0.2	0.829
**Triacylglycerole**	TG(17:1_38:6)	0.2±0.1	0.2±0.1	0.565	0.5±0.4	0.4±0.1	0.552
**Triacylglycerole**	TG(17:1_38:7)	0.1±0.1	0.1±0.1	0.852	0.1±0.1	0.1±0.1	0.909
**Triacylglycerole**	TG(17:2_34:2)	0.2±0.1	0.3±0.2	0.195	0.3±0.3	0.2±0.1	0.659
**Triacylglycerole**	TG(17:2_36:2)	0.3±0.1	0.5±0.2	0.168	0.3±0.2	0.4±0.2	0.148
**Triacylglycerole**	TG(17:2_36:3)	0.3±0.1	0.4±0.2	0.17	0.4±0.3	0.4±0.2	0.909
**Triacylglycerole**	TG(17:2_36:4)	0.3±0.1	0.5±0.2	0.161	0.5±0.4	0.5±0.2	0.892
**Triacylglycerole**	TG(17:2_38:5)	0.2±0.1	0.4±0.2	0.121	0.3±0.3	0.3±0.2	0.685
**Triacylglycerole**	TG(17:2_38:6)	0.3±0.1	0.3±0.1	0.575	0.3±0.2	0.4±0.2	0.411
**Triacylglycerole**	TG(17:2_38:7)	0.1±0.1	0.2±0.1	0.082	0.2±0.1	0.1±0.1	0.16
**Triacylglycerole**	TG(18:0_30:0)	5.3±1.5	12.9±11.7	0.302	10.6±9.1	12.9±6.4	0.731
**Triacylglycerole**	TG(18:0_30:1)	15.8±7.2	52.1±53.8	0.291	15.3±6.7	38.3±40	0.364
**Triacylglycerole**	TG(18:0_32:0)	2.3±1	5.6±4.6	0.264	4.5±4	5±2.2	0.852
**Triacylglycerole**	TG(18:0_32:1)	9.4±3.7	26.4±25.6	0.298	13.2±10.6	20.1±15.5	0.545
**Triacylglycerole**	TG(18:0_32:2)	8.5±3.2	22.1±22	0.329	16.2±14.6	23.1±16.5	0.612
**Triacylglycerole**	TG(18:0_34:2)	10.1±2.6	26.3±24.6	0.299	18±17.4	19.2±10.1	0.924
**Triacylglycerole**	TG(18:0_34:3)	3.3±1.1	7.4±4.5	0.174	4.6±4	4.3±2.4	0.923
**Triacylglycerole**	TG(18:0_36:1)	1.4±0.5	4.7±4.5	0.255	2.4±1.2	3.2±2.1	0.59
**Triacylglycerole**	TG(18:0_36:2)	7.2±2.6	23.3±24.1	0.294	11.2±8.1	14.5±11.1	0.685

**Triacylglycerole**	TG(18:0_36:3)	9.6±3	25.2±24.9	0.32	20±18.2	21.4±14	0.916
**Triacylglycerole**	TG(18:0_36:4)	5.3±1.3	10±6.9	0.286	13.8±13.9	12.3±5	0.867
**Triacylglycerole**	TG(18:0_36:5)	1.1±0.3	1.5±0.5	0.196	2.1±1.9	1.9±0.9	0.906
**Triacylglycerole**	TG(18:0_38:6)	2±0.4	2.3±0.6	0.39	4.6±4.1	3.9±1.6	0.794
**Triacylglycerole**	TG(18:0_38:7)	0.4±0.1	0.5±0.3	0.363	0.8±0.7	0.7±0.3	0.934
**Triacylglycerole**	TG(18:1_26:0)	119.5±55.4	269±182	0.223	100.4±48	192.9±177.8	0.418
**Triacylglycerole**	TG(18:1_28:1)	66.2±30.9	140.6±54.6	0.086	17.9±9.7	41.3±38.2	0.342
**Triacylglycerole**	TG(18:1_30:0)	91.7±34.2	218.9±153.4	0.211	143.9±140.2	137.6±95.4	0.951
**Triacylglycerole**	TG(18:1_30:1)	255.8±113.6	586.8±351.1	0.172	123.7±62.5	267.7±282	0.422
**Triacylglycerole**	TG(18:1_30:2)	123.2±59.4	291.2±203.8	0.22	78.8±37.3	176.4±181.6	0.397
**Triacylglycerole**	TG(18:1_32:0)	57.6±18	139.6±100	0.212	104.1±105.4	75±40.4	0.672
**Triacylglycerole**	TG(18:1_32:1)	153.2±60.5	376.3±231.6	0.158	119.1±92.8	146.3±122.5	0.77
**Triacylglycerole**	TG(18:1_32:2)	84.1±33.7	207±130.5	0.165	100.9±93.1	113.4±88.2	0.872
**Triacylglycerole**	TG(18:1_32:3)	8±3.2	17.7±7.8	0.092	9.3±7.8	11.1±6.2	0.762
**Triacylglycerole**	TG(18:1_33:0)	0.9±0.2	2±1.4	0.204	1.4±1.3	1.1±0.7	0.761
**Triacylglycerole**	TG(18:1_33:1)	2.4±0.8	6.2±3.2	0.087	3.8±3.4	2.8±1.5	0.643
**Triacylglycerole**	TG(18:1_33:2)	1.6±0.6	3.1±1.4	0.127	2.6±2.8	1.9±0.7	0.683
**Triacylglycerole**	TG(18:1_33:3)	0.3±0.2	0.5±0.2	0.037*§	0.6±0.5	0.4±0.1	0.483
**Triacylglycerole**	TG(18:1_34:1)	158.8±47.4	401.8±260.4	0.163	146.6±128.4	143.7±100.9	0.977
**Triacylglycerole**	TG(18:1_34:2)	158.6±55.4	387±216.2	0.127	166.3±164.6	143.8±92.2	0.844
**Triacylglycerole**	TG(18:1_34:3)	40.7±15.7	89.6±37	0.08	33.2±30.3	29.5±17.4	0.863
**Triacylglycerole**	TG(18:1_34:4)	3.6±1.2	6.6±1.5	0.032*§	5.7±5.3	5.5±2.5	0.959
**Triacylglycerole**	TG(18:1_35:2)	2.2±0.8	4.8±2.9	0.181	2±1.6	2±1.2	0.968
**Triacylglycerole**	TG(18:1_35:3)	1±0.4	2.1±1.1	0.143	1.3±1.2	1.1±0.7	0.831
**Triacylglycerole**	TG(18:1_36:0)	2.2±0.7	6.1±5.4	0.264	3±1.9	3.7±1.9	0.677
**Triacylglycerole**	TG(18:1_36:1)	21.4±7.7	64±54.4	0.228	22.3±16	28.1±24.4	0.742
**Triacylglycerole**	TG(18:1_36:2)	103.6±40.7	293.5±199.6	0.158	86.3±64	113.9±101.2	0.705
**Triacylglycerole**	TG(18:1_36:3)	107.1±42.4	267.3±181.2	0.187	134.8±121	130.8±100.5	0.967
**Triacylglycerole**	TG(18:1_36:4)	30.2±10.2	65±39.5	0.191	68.9±74.4	51.5±25.9	0.716
**Triacylglycerole**	TG(18:1_36:5)	3.7±0.9	6.7±2.3	0.081	9.5±10.1	7.3±3.2	0.736
**Triacylglycerole**	TG(18:1_36:6)	2±0.8	2.2±1.1	0.785	6.4±6	5.6±2.7	0.846
**Triacylglycerole**	TG(18:1_38:5)	5.2±1	7.1±1.3	0.094	11.4±10.7	9.1±3.6	0.733
**Triacylglycerole**	TG(18:1_38:6)	4.7±0.8	5.3±1.4	0.538	14.3±13.8	10.6±4.6	0.677
**Triacylglycerole**	TG(18:1_38:7)	0.8±0.3	1.2±0.5	0.212	1.6±1.4	1.4±0.6	0.822
**Triacylglycerole**	TG(18:2_28:0)	119.2±53.8	273.8±199.6	0.243	170.9±144.5	221.1±158	0.699
**Triacylglycerole**	TG(18:2_30:0)	73.3±26	170.9±127.4	0.242	190.9±210.6	159.3±91.2	0.82
**Triacylglycerole**	TG(18:2_30:1)	123.5±57.6	281.5±183.4	0.205	91.1±48.5	174.9±168.8	0.44
**Triacylglycerole**	TG(18:2_32:0)	49.9±15.2	110.6±81.2	0.251	148.6±175.2	89.5±40.7	0.591
**Triacylglycerole**	TG(18:2_32:1)	82.4±31.3	190.1±118.2	0.178	120.7±121.7	110.9±76.6	0.911

**Triacylglycerole**	TG(18:2_32:2)	35.8±13.8	80.3±47.9	0.173	97.1±112.1	77.8±40.1	0.789
**Triacylglycerole**	TG(18:2_33:0)	0.8±0.1	1.8±1.5	0.241	1.8±1.9	1.3±0.8	0.669
**Triacylglycerole**	TG(18:2_33:1)	1.7±0.5	3.6±2.3	0.203	4.1±4.2	2.7±1.2	0.594
**Triacylglycerole**	TG(18:2_33:2)	0.8±0.3	1.3±0.5	0.223	3.2±3.9	1.4±0.6	0.473
**Triacylglycerole**	TG(18:2_34:0)	14.2±3.4	34±28.7	0.282	29.3±30.7	25.4±11.9	0.846
**Triacylglycerole**	TG(18:2_34:1)	88.8±25.4	211.7±149.3	0.21	159.8±175.3	116±61.9	0.698
**Triacylglycerole**	TG(18:2_34:2)	69.7±23.1	144.4±74.7	0.149	162.6±197.8	101.4±45.4	0.621
**Triacylglycerole**	TG(18:2_34:3)	15±5.1	29.2±9.2	0.058	27.5±31.4	19.2±8.7	0.674
**Triacylglycerole**	TG(18:2_34:4)	2.4±0.7	3.3±0.7	0.136	5.8±5.8	5.6±2.7	0.966
**Triacylglycerole**	TG(18:2_35:1)	1±0.6	2.2±1.9	0.315	1.7±1.6	1.4±0.7	0.761
**Triacylglycerole**	TG(18:2_35:2)	1±0.4	2.1±1	0.107	2.4±2.5	1.4±0.6	0.543
**Triacylglycerole**	TG(18:2_35:3)	0.5±0.2	0.9±0.6	0.27	1.3±1.5	1±0.4	0.686
**Triacylglycerole**	TG(18:2_36:0)	2.2±0.6	4.9±4.6	0.343	3.7±3.3	4.4±2	0.779
**Triacylglycerole**	TG(18:2_36:1)	13.7±4.6	35.2±30.1	0.266	25.1±22.9	25.2±15.6	0.994
**Triacylglycerole**	TG(18:2_36:2)	55.4±20.8	133.8±90.2	0.193	86.6±85.4	78.6±48.7	0.894
**Triacylglycerole**	TG(18:2_36:3)	40.9±14.3	90.2±57.9	0.202	109.6±123.9	81.3±40.4	0.72
**Triacylglycerole**	TG(18:2_36:4)	14.2±3.9	22.8±9.2	0.185	63.6±76.4	40.8±18	0.633
**Triacylglycerole**	TG(18:2_36:5)	2.3±0.9	3±0.7	0.246	8.6±9.6	6.5±3.5	0.727
**Triacylglycerole**	TG(18:2_38:4)	4.7±0.9	5.8±1.2	0.221	11.5±11	9.7±3.7	0.786
**Triacylglycerole**	TG(18:2_38:5)	4.4±1.2	5±1	0.551	12.4±12.5	10.1±4.9	0.778
**Triacylglycerole**	TG(18:2_38:6)	4.3±1.1	3.7±1.2	0.59	14.1±14.5	11.7±6	0.793
**Triacylglycerole**	TG(18:3_30:0)	5.1±1.6	9±4.1	0.17	15.7±17.6	10.9±5.1	0.666
**Triacylglycerole**	TG(18:3_32:0)	3.4±1.1	5.5±2.1	0.167	11.1±12.6	6.4±2.6	0.548
**Triacylglycerole**	TG(18:3_32:1)	5.5±2	10.4±4.4	0.125	10.5±11.1	8.4±4.5	0.772
**Triacylglycerole**	TG(18:3_34:0)	1.3±0.2	2±1	0.246	2.5±2.6	2±0.9	0.749
**Triacylglycerole**	TG(18:3_34:1)	6.6±1.8	12.2±5.3	0.13	14.8±16.7	9.3±4.2	0.598
**Triacylglycerole**	TG(18:3_34:2)	4.8±1.3	8.8±2.8	0.062	12.2±14.1	7.3±3.4	0.583
**Triacylglycerole**	TG(18:3_34:3)	1.2±0.3	1.9±0.4	0.033*§	2.1±2.1	1.5±0.6	0.651
**Triacylglycerole**	TG(18:3_35:2)	0.2±0.1	0.2±0.1	0.096	0.3±0.2	0.2±0.1	0.702
**Triacylglycerole**	TG(18:3_36:1)	1.2±0.4	2.3±1.3	0.22	2.2±2	2.1±1.1	0.987
**Triacylglycerole**	TG(18:3_36:2)	4.1±1.1	8.3±4.5	0.157	7.1±6.8	6±3.4	0.818
**Triacylglycerole**	TG(18:3_36:3)	3±0.9	5.7±2.8	0.153	7.9±8.8	5.6±2.7	0.679
**Triacylglycerole**	TG(18:3_36:4)	1.2±0.4	2±0.6	0.061	4.3±4.8	2.9±1.3	0.62
**Triacylglycerole**	TG(18:3_38:5)	0.5±0.2	0.6±0.2	0.374	1±0.9	1±0.4	0.943
**Triacylglycerole**	TG(18:3_38:6)	0.5±0.2	0.5±0.2	0.714	1.4±1.3	1.2±0.6	0.874
**Triacylglycerole**	TG(20:0_32:3)	0.4±0.2	0.8±0.4	0.144	0.7±0.7	0.7±0.3	0.896
**Triacylglycerole**	TG(20:0_32:4)	0.4±0.2	0.6±0.3	0.244	1±1.2	0.6±0.3	0.622
**Triacylglycerole**	TG(20:1_30:1)	3.9±1.7	11±10.6	0.292	2.9±1.4	4.8±4.3	0.492
**Triacylglycerole**	TG(20:1_32:1)	2.9±1	7.1±6	0.262	2.7±1.8	3.1±2	0.802

**Triacylglycerole**	TG(20:1_32:2)	2.1±0.8	5.6±5.1	0.278	2.9±2.3	3.2±1.8	0.834
**Triacylglycerole**	TG(20:1_32:3)	0.4±0.1	0.8±0.4	0.121	0.5±0.5	0.6±0.3	0.873
**Triacylglycerole**	TG(20:1_34:0)	0.6±0.2	1±0.6	0.238	0.7±0.5	0.8±0.3	0.655
**Triacylglycerole**	TG(20:1_34:1)	2.6±0.9	6.1±4.1	0.194	2.8±2.3	2.8±1.3	0.999
**Triacylglycerole**	TG(20:1_34:2)	2.7±1	6.7±5.2	0.229	3.5±3.4	3.3±1.5	0.947
**Triacylglycerole**	TG(20:1_34:3)	0.9±0.3	1.8±0.9	0.133	1±0.9	0.9±0.4	0.89
**Triacylglycerole**	TG(20:2_32:0)	0.8±0.2	1.5±0.4	0.031*[§]	3.3±3.8	1.9±0.9	0.549
**Triacylglycerole**	TG(20:2_32:1)	1.3±0.4	2.7±1.5	0.17	3.2±3.6	2.6±1.1	0.789
**Triacylglycerole**	TG(20:2_34:1)	1.5±0.2	2.7±1.2	0.127	3.9±4.2	2.4±0.9	0.551
**Triacylglycerole**	TG(20:2_34:2)	1.5±0.4	2.5±1.2	0.21	4.7±5.8	2.6±1.1	0.565
**Triacylglycerole**	TG(20:2_34:3)	0.6±0.3	0.7±0.3	0.619	1.2±1.2	0.9±0.5	0.736
**Triacylglycerole**	TG(20:2_34:4)	0.1±0.1	0.1±0.1	0.148	0.3±0.3	0.2±0.2	0.822
**Triacylglycerole**	TG(20:2_36:5)	0.2±0.1	0.2±0.1	0.617	0.4±0.4	0.3±0.2	0.876
**Triacylglycerole**	TG(20:3_32:0)	2.7±0.9	3.4±0.6	0.336	6.6±6.7	4.7±2.2	0.65
**Triacylglycerole**	TG(20:3_32:1)	3.3±1	5.1±1.4	0.11	5.9±5.2	5.5±2.6	0.917
**Triacylglycerole**	TG(20:3_32:2)	2.6±0.8	3.7±0.9	0.14	5.6±5.4	5.9±3.4	0.932
**Triacylglycerole**	TG(20:3_34:0)	1.1±0.2	1.5±0.4	0.126	1.8±1.6	1.5±0.8	0.838
**Triacylglycerole**	TG(20:3_34:1)	3.7±0.8	5.6±1.4	0.073	7.1±6.6	5.4±2.4	0.693
**Triacylglycerole**	TG(20:3_34:2)	3.2±0.8	5.5±1.4	0.04*[§]	7.4±7.8	5.8±3	0.755
**Triacylglycerole**	TG(20:3_34:3)	1.1±0.5	1.6±0.5	0.144	1.9±1.8	1.5±0.8	0.692
**Triacylglycerole**	TG(20:3_36:3)	2.4±0.6	3.7±1	0.091	5.5±5.1	4.7±2.2	0.823
**Triacylglycerole**	TG(20:3_36:4)	1.6±0.4	1.6±0.4	0.802	3.7±4	3.3±2	0.875
**Triacylglycerole**	TG(20:3_36:5)	0.3±0.2	0.3±0.2	0.887	0.6±0.5	0.7±0.4	0.76
**Triacylglycerole**	TG(20:4_30:0)	14.9±4.8	19.3±4.7	0.294	24.9±17.6	26±11	0.93
**Triacylglycerole**	TG(20:4_32:0)	12.8±2.8	15.3±2.5	0.287	19.6±14.1	17.6±7.9	0.838
**Triacylglycerole**	TG(20:4_32:1)	8.9±2.2	12.9±2.9	0.107	10.6±6.8	11.6±4.5	0.835
**Triacylglycerole**	TG(20:4_32:2)	6.1±1.7	7.6±1.8	0.308	9.5±7	12.2±5.3	0.609
**Triacylglycerole**	TG(20:4_33:2)	0.3±0.1	0.3±0.1	0.913	0.6±0.4	0.4±0.2	0.445
**Triacylglycerole**	TG(20:4_34:0)	5.4±1.1	6.5±1.1	0.223	7.4±5	7.5±3.2	0.973
**Triacylglycerole**	TG(20:4_34:1)	14.2±1.8	18.8±1.7	0.016*	17.8±12.4	17.1±6.3	0.938
**Triacylglycerole**	TG(20:4_34:2)	12.6±1.9	16.8±2.4	0.048*	18.8±14.5	18.6±7.5	0.986
**Triacylglycerole**	TG(20:4_34:3)	2.9±0.7	3.8±1	0.244	3±2.1	3.4±1.5	0.818
**Triacylglycerole**	TG(20:4_35:3)	0.2±0.1	0.2±0.1	0.429	0.3±0.2	0.2±0.1	0.363
**Triacylglycerole**	TG(20:4_36:2)	8.8±1.6	11.4±2	0.118	13.4±10.3	13±4.9	0.957
**Triacylglycerole**	TG(20:4_36:3)	7.4±1.6	9.3±1.8	0.207	10.8±8	12.1±5.2	0.821
**Triacylglycerole**	TG(20:4_36:4)	6.5±0.9	7.5±1	0.219	8.4±5.2	10.2±4.4	0.673
**Triacylglycerole**	TG(20:4_36:5)	1.1±0.3	1.2±0.5	0.6	1.2±0.7	1.8±0.8	0.313
**Triacylglycerole**	TG(20:5_34:0)	0.3±0.1	0.4±0.1	0.245	0.5±0.5	0.6±0.3	0.79
**Triacylglycerole**	TG(20:5_34:1)	1.4±0.4	1.5±0.2	0.624	2.5±1.9	2.4±1.1	0.932

**Triacylglycerole**	TG(20:5_34:2)	1±0.3	1.1±0.4	0.55	2.4±2.3	2.4±1.4	0.98
**Triacylglycerole**	TG(20:5_36:2)	0.9±0.1	1±0.3	0.357	1.3±0.9	1.4±0.5	0.792
**Triacylglycerole**	TG(20:5_36:3)	0.7±0.3	0.7±0.1	0.68	1.4±1.2	1.4±0.8	0.962
**Triacylglycerole**	TG(22:0_32:4)	0.1±0.1	0.1±0.1	0.345	0.1±0.1	0.1±0.1	0.862
**Triacylglycerole**	TG(22:2_32:4)	0.2±0.1	0.3±0.2	0.299	0.4±0.4	0.5±0.2	0.935
**Triacylglycerole**	TG(22:4_32:0)	1.2±0.3	1.6±0.2	0.105	2.2±1.5	2.3±1.1	0.896
**Triacylglycerole**	TG(22:4_32:2)	0.6±0.2	0.7±0.2	0.767	1.1±0.8	1.4±0.7	0.578
**Triacylglycerole**	TG(22:4_34:2)	1.4±0.5	2±0.4	0.147	2.5±1.9	2.8±1.2	0.833
**Triacylglycerole**	TG(22:5_32:0)	3.1±0.9	3.3±0.4	0.769	6.2±5	5.7±2.4	0.878
**Triacylglycerole**	TG(22:5_32:1)	2.6±0.8	3.2±1	0.41	5.3±4.3	5.1±2	0.928
**Triacylglycerole**	TG(22:5_34:1)	3.1±0.5	4±0.5	0.062	7.7±6.7	6±2.1	0.698
**Triacylglycerole**	TG(22:5_34:2)	2.8±0.7	3.6±0.6	0.178	8.6±8.4	6.4±3.1	0.685
**Triacylglycerole**	TG(22:5_34:3)	0.9±0.3	1±0.4	0.671	1.9±1.6	1.9±0.8	0.987
**Triacylglycerole**	TG(22:6_32:0)	16.6±5.5	15.9±5.1	0.876	51.6±49.3	43.2±20.7	0.793
**Triacylglycerole**	TG(22:6_32:1)	12.1±3.9	12.6±7.2	0.926	34.4±32.8	28.6±13.7	0.787
**Triacylglycerole**	TG(22:6_34:1)	14.7±3.8	14.3±5.5	0.935	51.7±51.9	37.3±17.6	0.666
**Triacylglycerole**	TG(22:6_34:2)	12.7±3.5	11.8±4.9	0.809	50.5±52.3	39.8±21.3	0.756
**Triacylglycerole**	TG(22:6_34:3)	3±1.1	3±2.1	0.993	8.8±8.2	7.9±4.5	0.873

Statistical analyses were performed with student's *t*-Test. n=4-6 per group, all data are mean ± SD. Concentrations were for amino acids presented as nmol/mg tissue, all other metabolites are presented in pmol/mg tissue. Significance <0.05 are marked with *, FC >1.5 are marked with § (if significant <0.05). B.dl. = below detection limit, n.a.= not assessed.
