# Supplementary material for: Carnosinase-1 Knock-Out Reduces Kidney Fibrosis in Type-1 Diabetic Mice on High Fat Diet
Source: Antioxidants (Basel). 2023 Jun 14;12(6):1270. doi: 10.3390/antiox12061270 (PMC10295340; doi:10.3390/antiox12061270)
Supplement: Supplementary file 1 [file antioxidants-12-01270-s001.zip › Suppl. Tables 1-8.pdf]

Suppl. Table 1: Insulin injection scale

Blood sugar (mg/dl)	Insulin injection in units
**200 - 349**	0
**350 - 400**	1
**401 - 460**	2
**461 - 510**	3
**511 - 600**	4
**≥ 600**	5

Suppl. Table 2: Aperio ImageScope settings

	AGE	phospho-Smad2/3	Sirius red	TSP-1	phospho-NRF2
**view width**	1000	1000	1000	1000	1000
**view height**	1000	1000	1000	1000	1000
**overlap size**	0	0	0	0	0
**image zoom**	1	1	1	1	1
**markup compression type**	same as processed image	same as processed image	same as processed image	same as processed image	same as processed image
**compression quality**	30	30	30	30	30
**classifier neighbourhood**	0	0	0	0	0
**classifier**	none	none	none	none	none
**hue value**	0.1	0.1	0	0.1	0.1
**hue width**	0.6	0.6	0.15	0.1	0.04
**color saturation threshold**	0.03	0.03	0.03	0.1	0.1
**Iwp (high)**	220	220	220	220	220
**Iwp (low) = Isp (high)**	190	190	175	190	190
**Ip (low) = Isp (high)**	120	120	100	120	120
**Isp (low)**	0	0	0	0	0
**Inp (high)**	-1	-1	-1	-1	-2

Lwp (high) = upper limit of total intensity of weak positive pixels, Lwp (low) = lower limit of total intensity of weak positive positive, lsp (high) = higher limit of total intensity of strong positive pixels, lsp (low) = lower limit of total intensity of strong positive pixels, Ip (low) = lower limit of total intensity of positive pixels, Inp (high) = higher limit of of total intensity of negative pixels

Suppl. Table 3: Oligonucleotides for qRT-PCR evaluations

**Oligonucleotides:**		
**Gene:**	Orientation:	sequence from 5' - 3'
eNOS	forward	GGTCCCTCATGCCAATCTCT
	reverse	AGAACTCTTCACTCTGCCCC
FAS	forward	GAGGCCCATTTTGCTGTCAA
	reverse	CAGGGTGCAGTTTGTTTCCA
Fibronectin-1	forward	TCCCGGGCAGAAAGTACATT
	reverse	TTCAGGGAGGTTGAGCTCTG
Glu-Cys-ligase	forward	GGGGTGACGAGGTGGAGTA
	reverse	GTTGGGGTTTGTCCTCTCCC
Ho-1	forward	TTCAGAAGGGTCAGGTGTCC
	reverse	CAGTGAGGCCCATACCAGAA
Hsf1	forward	CTACGCCCGATCAGACGTTT
	reverse	GGCTGGAGATGGAGCTGAGTA
Hspa1	forward	GAGATCGACTCTCTGTTCGAGG
	reverse	GCCCGTTGAAGAAGTCCTG
Hspa1a	forward	TGGTGCAGTCCGACATGAAG
	reverse	GCTGAGAGTCGTTGAAGTAGGC
Hspa1b	forward	GAGATCGACTCTCTGTTCGAGG
	reverse	GCCCGTTGAAGAAGTCCTG
HspA8	forward	TCTCGGCACCACCTACTCC
	reverse	CTACGCCCGATCAGACGTTT
iNOS	forward	AATCTTGGAGCGAGTTGTGG
	reverse	CAGGAAGTAGGTGAGGGCTTG
Nfe2l2	forward	CGAGATATACGCAGGAGAGGTAAGA
	reverse	GCTCGACAATGTTCTCCAGCTT
NfκB	forward	GAAATTCCTGATCCAGACAAAAAG
	reverse	ATCACTTCAATGGCCTCTGTGTAG
p53	forward	CCCCTGTCATCTTTTGTCCCT
	reverse	AGCTGGCAGAATAGCTTATTGAG
Ptgs2	forward	TTCGTCGTCTCGTTGCAAAG
	reverse	CCCAGGTCCTCGCTTATGAT
Renin	forward	AGAACACCGTCAAACTTGGC
	reverse	CCTCTACCTTGCTTGTGGGA
Sirtuin-1	forward	AACATCGCAGTCTCCAAGGA
	reverse	TGCCATCATGAAGCCAGAGA
Tfn-α	forward	AATGGCCTCCCTCTCATCAGTT
	reverse	CCACTTGGTGGTTTGCTACGA
Tgf-β	forward	CAACAATTCCTGGCGTTACCTTGG
	reverse	GAAAGCCCTGTATTCCGTCTCCTT
Vegf	forward	CGCTCCAGGATTTAAACCGG
	reverse	GCTGTAACGATGAAGCCCTG
VegfR	forward	TGAATGTCCCACCCCAGATC
	reverse	CCTCCACGTGTCTCCATTCT
Wisp1	forward	CAGCACCACTAGAGGAAACGA
	reverse	CTGGGCACATATCTTACAGCATT

Suppl. Table 4: Body weight

4.1 Normal diet

weeks of experiment	WT	*Cndp1*-KO	***p*** (vs WT)	WT STZ	***p*** (vs WT)	*Cndp1*-KO STZ	***p*** (vs WT STZ)
**0**	19.6±1.0	19.6±1.8	0.999	19.0±1.1	0.289	19.0±1.4	0.999
**12**	23.2±1.3	24.6±2.3	0.339	19.8±1.8	<0.001	18.8±2.8	0.424
**16**	22.8±1.4	25.8±3.1	0.057	18.9±2.4	<0.001	19.7±2.6	0.674
**20**	24.1±1.7	27.1±3.5	0.115	19.3±2.2	<0.001	19.8±2.8	0.868
**24**	24.2±1.9	27.0±3.3	0.134	19.6±2.3	<0.001	19.7±2.8	0.999
**28**	24.7±1.2	27.9±3.9	0.110	19.6±2.5	<0.001	20.3±2.6	0.819
**30**	24.3±1.8	28.5±4.1	0.044	19.9±2.3	0.001	20.2±2.7	0.989
**32**	23.9±1.6	27.8±4.4	0.086	19.5±2.1	<0.001	19.9±3.1	0.950
**average**	23.3±1.5	26.0±2.7	0.017	19.4±.03	0.001	19.6±0.5	0.993

4.2 High fat diet

weeks of experiment	WT HFD	*Cndp1*-KO HFD	***p*** (vs WT HFD)	***p*** (vs *WT ND*)	***p*** (vs *Cndp1*-KO ND)	WT HFD + STZ	***p*** (vs WT HFD)	*Cndp1*-KO HFD + STZ	***p*** (vs WT HFD STZ)
**0**	19.8±1.7	19.1±1.5	0.667	0.986	0.9915	18.7±1.4	0.240	19.8±2.0	0.118
**12**	32.6±5.0	35.2±6.1	0.616	0.116	<0.001	19.4±1.9	<0.001	21.2±1.8	0.010
**16**	36.1±5.3	39.8±6.2	0.357	0.010	<0.001	20.8±2.0	<0.001	22.0±2.7	0.304
**20**	39.5±4.6	42.5±6.4	0.465	0.002	<0.001	20.7±1.7	<0.001	21.9±2.7	0.280
**24**	41.6±4.8	45.5±7.5	0.320	0.001	<0.001	20.9±1.7	<0.001	22.3±1.9	0.055
**28**	45.5±5.3	49.2±7.4	0.406	<0.001	<0.001	21.3±1.7	<0.001	22.4±2.6	0.479
**30**	43.5±5.4	49.5±7.4	0.082	<0.001	<0.001	21.5±1.9	<0.001	23.4±2.3	0.059
**32**	42.8±4.8	48.6±7.9	0.083	<0.001	<0.001	21.7±2.7	<0.001	23.0±2.2	0.337
**average**	37.7±7.1	41.1±9.6	0.723	<0.001	<0.001	20.6±0.9	0.001	22.0±1.0	0.975

Statistical analyses were performed with mixed effect models. n=10-29 per group. Data are mean ± SD gram body weight.

Suppl. Table 5: Energy intake

5.1 Normal diet

weeks of experiment	WT	*Cndp1*-KO	*p* (vs WT)	WT STZ	*p* (vs WT)	*Cndp1*-KO STZ	*p* (vs WT STZ)
**12**	36.4±3.1	41.1±8.7	0.616	53.6±15.9	0.008	53.6±16.4	1.000
**16**	37.4±3.8	35.9±8.1	0.975	54.9±9.6	0.001	54.1±13.3	0.998
**20**	33.5±6.1	39.6±5.6	0.364	50.7±8.8	0.004	58.5±15.3	0.351
**24**	35.7±3.4	45.2±5.7	0.035	46.8±11.0	0.039	57.5±8.9	0.080
**28**	37.2±5.9	43.0±7.2	0.492	49.4±11.4	0.072	52.1±9.7	0.933
**Average**	36.0±4.4	40.9±7.0	0.323	51.0±11.3	<0.001	55.1±12.7	0.133

5.2 High fat diet

weeks of experiment	WT HFD	*Cndp1*-KO HFD	*p* (vs WT HFD)	WT HFD STZ	*p* (vs WT HFD)	*Cndp1*-KO HFD STZ	*p* (vs WT HFD STZ)
**12**	30.9±5.4	40.7±9.6	0.17	60.2±12.9	0.001	60.7±17.2	0.999
**16**	56.8±6.4	48.6±9.4	0.341	52.7±7.6	0.712	54.2±8.5	0.998
**20**	33.7±12.2	47±16.9	0.451	56.5±10.3	0.078	62.7±12.7	0.351
**24**	37.2±6.8	40.2±7.4	0.890	54.7±7.6	0.020	59.1±14.0	0.080
**28**	37.1±14.0	49.4±9.3	0.468	57.1±9.2	0.172	65.1±8.2	0.933
**average**	39.6±7.6	44.1±10.8	0.543	56.0±9.6	<0.001	59.1±13.0	0.501

Statistical analyses were performed with mixed effect model, n=6-16 per group. Data are mean ± SD in kJ/mouse/24h.

Suppl. Table 6: Liquid intake

6.1 Normal diet

weeks of experiment	WT	*Cndp1*-KO	*p* (vs WT)	WT STZ	*p* (vs WT)	*Cndp1*-KO STZ	*p* (vs WT STZ)
**12**	4.2±0.2	4.9±1.2	0.492	17.8±4.5	<0.001	18.6±6.7	0.976
**16**	4.3±0.5	4.5±0.8	0.936	19.2±4.0	<0.001	19.0±5.7	0.999
**20**	4.1±0.5	4.6±0.7	0.354	17.4±4.3	<0.001	21.3±6.3	0.249
**24**	4.5±0.3	4.9±0.7	0.429	14.8±3.7	<0.001	17.7±3.7	0.294
**28**	4.3±0.2	4.5±0.8	0.921	16.5±4.9	0.001	19.1±5.2	0.653
**average**	4.2±0.3	4.6±0.8	0.983	17.1±4.2	<0.001	19.1±5.5	0.041

6.2 High fat diet

weeks of experiment	WT HFD	*Cndp1*-KO HFD	*p* (vs WT HFD)	WT HFD STZ	*p* (vs WT HFD)	*Cndp1*-KO HFD STZ	*p* (vs WT HFD STZ)
**12**	2.8±0.6	3.4±0.8	0.476	12.3±2.7	<0.001	12.3±4.1	0.999
**16**	3.1±0.4	3.2±0.6	0.955	11.9±3.1	<0.001	9.6±3.3	0.281
**20**	2.4±1.0	3.0±0.8	0.713	12.1±3.7	<0.001	12.3±2.0	0.998
**24**	2.4±0.5	2.9±0.6	0.364	12.3±3.5	<0.001	12.6±2.3	0.992
**28**	2.4±0.5	3.1±0.7	0.190	11.8±5.2	0.001	13.1±3.4	0.895
**average**	2.6±0.5	3.1±0.7	0.904	12.0±3.6	<0.001	12.0±3.0	0.998

Statistical analyses were performed with mixed effect models. n=6-17 per group. Data are mean ± SD g water/mouse/24h.

Suppl. Table 7: Fasting blood glucose levels

7.1 Normal diet

weeks of experiment	WT	*Cndp1*-KO	*p* (vs WT)	WT STZ	*p* (vs WT)	*Cndp1*-KO STZ	*p* (vs WT STZ)
**12**	6.5±1	7.5±1.3	0.205	21.5±5	<0.001	20.2±5	0.792
**16**	6.3±0.5	7.4±1.2	0.073	21.6±4.8	<0.001	21.2±4.5	0.991
**20**	6.6±1.1	7.5±1.4	0.369	20.4±4.5	<0.001	22.9±4.5	0.206
**24**	7.2±1.0	6.6±2.2	0.878	21.3±5.9	<0.001	21.3±3.0	1.000
**28**	6.5±0.8	7.0±1.5	0.772	20.4±4.8	<0.001	19.6±4.2	0.935
**30**	7.5±1.4	6.9±0.9	0.599	19.4±4.9	<0.001	19.2±6.5	1.000
**32**	6.5±0.8	7.3±0.8	0.132	19.3±4.5	<0.001	20.6±3.1	0.751
**average**	6.7±0.4	7.1±0.4	0.745	20.5±1.0	<0.001	20.7±1.2	0.982

7.2 High fat diet

weeks of experiment	WT HFD	*Cndp1*-KO HFD	*p* (vs WT HFD)	WT HFD STZ	*p* (vs WT HFD)	*Cndp1*-KO HFD STZ	*p* (vs WT HFD STZ)
**12**	8.0±1.5	7.7±0.9	0.879	20.1±2.7	<0.001	19.8±3.0	0.994
**16**	7.9±1.0	8.4±1.5	0.672	21.5±3.1	<0.001	20.0±3.1	0.356
**20**	8.2±1.0	9.4±1.6	0.064	21.3±2.8	<0.001	20.7±2.6	0.899
**24**	9.5±1.4	8.6±1.8	0.451	21.9±3.9	<0.001	20.7±3.4	0.729
**28**	8.8±1.5	8.1±1.4	0.684	22.7±4.3	<0.001	21.0±3.6	0.568
**30**	8.3±1.2	8.4±1.0	0.987	21.4±4.1	<0.001	21.6±4.6	0.999
**32**	8.7±1.2	8.8±1.1	0.972	20.8±4.0	<0.001	20.2±3.6	0.961
**average**	8.4±0.5	8.5±0.6	0.999	21.3±0.8	<0.001	20.6±0.6	0.125

Statistical analyses were performed with mixed effect model. n=10-27 per group. Data are mean ± SD in Blood glucose levels in mmol/l.

Suppl. Table 8: Kidney protein expression of HSP70 protein family members, carnosine synthase 1, phospho-NRF2 and thrombospondin-1

8.1 Normal diet

Protein	WT	*Cndp1*-KO	*p* (vs WT)	WT STZ	*p* (vs WT)	*Cndp1*-KO STZ	*p* (vs WT STZ)
**HSPA1A/A1B**	1.0±0.6	0.9±0.6	0.999	1.1±0.6	0.999	1.9±2.0	0.774
**HSPA9**	1.0±0.3	1.0±0.4	1.000	1.3±0.4	0.815	1.2±0.2	0.994
**HSP60**	1.0±1.1	1.6±1.4	0.841	0.7±0.4	0.970	0.7±0.3	0.999
**HSF1**	1.0±0.4	1.1±0.1	1.000	2.0±1.6	0.493	1.6±0.5	0.966
**Carnosine Synthase 1**	0.6±0.2	0.4±0.3	0.443	0.3±0.2	0.107	0.3±0.3	1.000
**Phospho-NRF2**	0.4±0.1	0.3±0.2	0.403	0.3±0.1	0.911	0.3±0.1	0.403
**Thrombospondin-1**	1.0±0.6	1.1±1.1	0.994	0.9±0.6	0.981	1.2±0.9	0.818

8.2. High fat diet

Protein	WT HFD	*Cndp1*-KO HFD	*p* (vs WT HFD)	WT HFD STZ	*p* (vs WT HFD)	*Cndp1*-KO HFD STZ	*p* (vs WT HFD STZ)
**Carnosine synthase 1**	0.5±0.2	0.4±0.1	0.568	0.3±0.3	0.130	0.6±0.3	0.021
**Phospho-NRF2**	0.3±0.1	0.2±0.1	0.828	0.3±0.1	0.969	0.3±0.2	0.908
**Thrombospondin-1**	1.1±0.6	0.9±0.6	0.752	1.2±0.7	0.999	1.2±0.6	0.999

Statistical analyses were performed with one-way ANOVA. n=4 per group. Data are mean ± SD. For HSP-70 proteins, data were normalized to WT.
