# Supplementary material for: Carnosinase-1 Knock-Out Reduces Kidney Fibrosis in Type-1 Diabetic Mice on High Fat Diet
Source: Antioxidants (Basel). 2023 Jun 14;12(6):1270. doi: 10.3390/antiox12061270 (PMC10295340; doi:10.3390/antiox12061270)
Supplement: Supplementary file 1 [file antioxidants-12-01270-s001.zip › Suppl. Tables 10-13.pdf]

Suppl. Table 10: Kidney arteriolar lumen/vessel ratio

10.1 Normal diet

	WT	*Cndp1*-KO	*p* (vs WT)	WT STZ	*p* (vs WT)	*Cndp1*-KO STZ	*p* (vs WT STZ)
**Lumen/vessel ratio**	0.62±0.03	0.6±0.05	0.764	0.58±0.07	0.156	0.56±0.06	0.726
**Media thickness (% of vessel)**	14.1±1.40	14.2±1.80	0.990	16.1±2.90	0.132	15.9±1.90	0.987
**Intima thickness (% of vessel)**	5.74±1.26	7.3±1.28	0.132	6.48±2.00	0.702	8.05±1.56	0.069

10.2 High fat diet

	WT HFD	*Cndp1*-KO HFD	*p* (vs WT HFD)	WT HFD STZ	*p* (vs WT HFD)	*Cndp1*-KO HFD STZ	*p* (vs WT HFD STZ)
**Lumen/vessel ratio**	0.63±0.07	0.61±0.04	0.889	0.58±0.05	0.112	0.61±0.06	0.401
**Media thickness (% of vessel)**	13.4±2.30	14.4±1.20	0.430	15.7±1.7	0.004	14.6±1.60	0.192
**Intima thickness (% of vessel)**	6.37±1.64	5.47±2.28	0.620	6.84±1.14	0.926	6.72±2.26	0.990

Statistical analyses were performed with one-way ANOVA. n=10-20 per group. Data are mean ± SD.

Suppl. Table 11: Kidney advanced glycation end-product (AGE) and 4-hydroxynonal (HNE) abundance

10.1 Normal diet

	**WT**	***Cndp1*-KO**	***p* (vs WT)**	**WT STZ**	***p* (vs WT)**	***Cndp1*-KO STZ**	***p* (vs WT STZ)**
**AGEs**	1.1±0.1	1.0±0.6	0.971	1.8±0.5	0.032	1±0.6	0.009
**HNE**	3.6±2.9	10.3±4.2	0.15	15.7±7.4	0.005	3.3±1.6	0.006

10.2 High fat diet

	**WT HFD**	***Cndp1*-KO HFD**	***p* (vs WT HFD)**	**WT HFD STZ**	***p* (vs WT HFD)**	***Cndp1*-KO HFD STZ**	***p* (vs WT HFD STZ)**
**AGEs**	1.0±0.5	2.8±2.1	0.011	1.6±0.3	0.716	1.6±0.6	0.998
**HNE**	2.0±1.1	2.5±1.5	0.969	3.5±2.4	0.416	0.1±0.1	0.023

Statistical analyses were performed with one-way ANOVA. n=4-10 per group. Data are mean ± SD µg/mg protein. AGEs were determined densitometrical in tubular structure and normalized to WT for normal diet or WT HFD for high fat diet respectively. HNE was determined via ELISA in tissue lysate.

Suppl. Table 12: Kidney gene expression levels

12.1 Normal diet

Gen	WT	*Cndp1*-KO	***p*** **(vs WT)**	WT STZ	***p*** **(vs WT)**	*Cndp1*-KO STZ	***p*** **(vs WT STZ)**
eNOS	1.03±0.24	0.94±0.18	0.921	1.2±0.31	0.639	1.00±0.27	0.513
FAS	1.01±0.11	1.01±0.22	0.999	0.89±0.10	0.813	0.99±0.39	0.878
Fibronectin-1	1.03±0.27	0.94±0.27	0.930	1.22±0.24	0.685	1.22±0.32	0.999
Glu-Cys-ligase	1.02±0.22	1.02±0.41	0.999	0.95±0.22	0.815	1.26±1.11	0.815
Ho-1	1.04±0.31	1.44±0.52	0.366	1.37±0.43	0.530	1.55±0.29	0.868
Hsf1	1.03±0.27	1.04±0.15	0.999	1.73±0.35	0.132	1.87±0.70	0.967
Hsp70 a1	1.30±1.18	1.09±0.74	0.972	0.81±0.43	0.717	1.19±0.63	0.864
Hspa1a	1.01±0.17	0.71±0.26	0.348	0.84±0.33	0.759	0.78±0.23	0.986
Hspa1b	1.04±0.32	0.57±0.16	0.078	0.61±0.24	0.112	0.72±0.25	0.912
HspA8	1.01±0.11	1.18±0.15	0.908	2.09±0.36	0.006	1.91±0.61	0.893
iNOS	1.06±0.34	1.02±0.42	0.998	1.13±0.31	0.977	0.67±0.20	0.097
Nfe2l2	1.03±0.25	0.95±0.12	0.940	0.95±0.30	0.940	0.86±0.29	0.937
NfκB	1.02±0.21	1.10±0.22	0.935	1.19±0.18	0.582	1.12±0.27	0.956
p53	1.01±0.11	1.00±0.11	0.999	0.96±0.18	0.951	0.76±0.26	0.222
Ptgs2	1.59±1.74	0.79±0.63	0.635	0.88±0.47	0.681	1.15±1.01	0.978
Renin	1.06±0.42	1.89±1.09	0.194	1.27±0.65	0.951	0.93±0.37	0.826
Sirtuin-1	1.01±0.15	1.14±0.08	0.717	0.91±0.35	0.823	0.90±0.21	0.999
Tnf-α	1.09±0.53	0.77±0.44	0.897	1.39±1.20	0.913	1.47±0.81	0.999
Tgf-β	1.03±0.24	1.06±0.15	0.997	1.47±0.33	0.057	1.23±0.36	0.458
Vegf	1.02±0.21	0.96±0.24	0.958	0.92±0.24	0.853	0.74±0.20	0.578
VegfR	1.02±0.18	1.18±0.16	0.700	1.04±0.27	0.998	1.08±0.39	0.993
Wisp1	1.04±0.30	1.44±0.64	0.290	1.42±0.11	0.615	1.38±0.19	0.999

12.2 High fat diet

Gen	WT HFD	*Cndp1*-KO HFD	*p* (vs WT HFD)	WT HFD STZ	*p* (vs WT HFD)	*Cndp1*-KO HFD STZ	*p* (vs WT HFD + STZ)
eNOS	1.01±0.15	1.10±0.17	0.831	1.11±0.13	0.809	1.07±0.24	0.987
FAS	1.03±0.27	1.19±0.22	0.906	1.41±0.60	0.367	1.17±0.37	0.720
Fibronectin-1	1.03±0.25	1.01±0.21	0.999	1.34±0.85	0.685	1.28±0.34	0.997
Glu-Cys-ligase	1.02±0.20	0.98±0.24	0.999	1.15±0.73	0.953	0.90±0.28	0.735
Ho-1	1.06±0.44	1.19±0.32	0.954	1.52±0.66	0.301	1.14±0.12	0.483
Hsp70 a1	1.05±0.37	1.15±0.78	0.999	2.35±2.12	0.235	1.14±0.16	0.288
iNOS	1.01±0.13	1.08±0.43	0.987	1.24±0.44	0.676	1.10±0.32	0.900
Nfe2l2	1.01±0.15	0.98±0.17	0.981	1.16±0.12	0.364	0.82±0.18	0.005
NfκB	1.02±0.18	0.82±0.09	0.117	0.94±0.14	0.812	0.82±0.18	0.526
p53	1.02±0.16	1.06±0.20	0.981	1.20±0.25	0.321	0.98±0.14	0.182
Ptgs2	1.35±1.33	1.18±0.78	0.994	1.85±1.51	0.875	2.07±0.64	0.988
Renin	1.11±0.62	1.10±0.60	0.999	0.52±0.14	0.118	0.40±0.10	0.958
Sirtuin-1	1.02±0.23	1.05±0.16	0.995	0.68±0.22	0.028	0.66±0.17	0.999
Tnf-α	1.01±0.16	1.41±0.45	0.581	2.41±0.21	0.002	2.36±0.91	0.999
Tgf-β	1.01±0.15	1.03±0.11	0.990	1.59±0.37	0.002	1.29±0.21	0.134
Vegf	1.01±0.14	0.92±0.18	0.606	0.61±0.08	0.999	0.61±0.10	0.999
VegfR	1.01±0.15	1.28±0.36	0.370	0.84±0.26	0.710	1.02±0.32	0.692
Wisp1	1.02±0.17	1.18±0.24	0.807	1.26±0.45	0.382	1.41±0.40	0.868

Statistical analyses were performed with one-way ANOVA. N=5-6 per group. Data are mean ± SD. Data are $2^{-\Delta\Delta CT}$.

Suppl. Table 13: Urine albumin to creatinine ratios

13.1 Normal diet

**WT**	***Cndp1*-KO**	***p* (vs W)**	**WT STZ**	***p* (vs WT)**	***Cndp1*-KO STZ**	***p* (vs WT STZ)**
16.3±7.5	18.9±9.5	0.60	63.7±26.7	0.002	91.7±67.1	0.46

13.2 High fat diet

**WT HFD**	***Cndp1*-KO HFD**	***p* (vs WT HFD)**	**WT HFD STZ**	***p* (vs WT HFD)**	***Cndp1*-KO HFD STZ**	***p* (vs WT HFD + STZ)**
14.5±5.3	33.8±40.4	0.31	39.4±27.7	0.06	29.8±20.3	0.3

Urinary albumin to creatinine ratio (mg/g) at week 32.
